# Changes in the proteome of sea urchin *Paracentrotus lividus* coelomocytes in response to LPS injection into the body cavity

**DOI:** 10.1371/journal.pone.0228893

**Published:** 2020-02-19

**Authors:** Luigi Inguglia, Marco Chiaramonte, Vincenzo Arizza, Lilla Turiák, Károly Vékey, Laszlo Drahos, Rosa Pitonzo, Giuseppe Avellone, Vita Di Stefano

**Affiliations:** 1 Department STEBICEF, University of the Study of Palermo, Palermo, Italy; 2 MS Proteomics Research Group, Research Centre for Natural Sciences, Hungarian Academy of Sciences, Magyar tudósok körútja, Budapest, Hungary; Chang Gung University, TAIWAN

## Abstract

**Background:**

The immune system of echinoderm sea urchins is characterised by a high degree of complexity that is not completely understood. The Mediterranean sea urchin *Paracentrotus lividus* coelomocytes mediate immune responses through phagocytosis, encapsulation of non-self particles, and production of diffusible factors including antimicrobial molecules. Details of these processes, and molecular pathways driving these mechanisms, are still to be fully elucidated.

**Principal findings:**

In the present study we treated the sea urchin *P*. *lividus* with the bacterial lipopolysaccharide (LPS) and collected coelomocytes at different time-points (1, 3, 6 and 24 hours). We have shown, using label-free quantitative mass spectrometry, how LPS is able to modulate the coelomocyte proteome and to effect cellular pathways, such as endocytosis and phagocytosis, as soon as the immunomodulating agent is injected. The present study has also shown that treatment can modulate various cellular processes such as cytoskeleton reorganisation, and stress and energetic homeostasis.

**Conclusions:**

Our data demonstrates, through mass spectrometry and the following functional annotation bioinformatics analysis, how the bacterial wall constituent is sufficient to set off an immune response inducing cytoskeleton reorganisation, the appearance of clusters of heat shock proteins (Hsp) and histone proteins and the activation of the endocytic and phagocytic pathways. Data are available via ProteomeXchange with identifier PXD008439.

## Introduction

Despite the apparent simplicity of the body organization of echinoderms, and in particular that of sea urchins, their immune system is far from being well understood and is specialised to perform a variety of functions. In particular, the echinoderm immune cells are a heterogeneous population, both at the morphological and functional level. Their profile can vary between species in terms of morphology, abundance, size, role and physiology. Four subpopulations of immune cells, phagocytes, vibratile cells, colourless and red spherule cells [1,2], were described in *Strongylocentrotus purpuratus* (purple sea urchin) and in *Paracentrotus lividus* [3–5]. The coelomocytes, cells that circulate in the coelomic fluid, mediate immune responses through phagocytosis and encapsulation of non-self particles in addition to the production of antimicrobial molecules. These non-self molecules are known as pathogen-associated molecular patterns (PAMPs), and their receptors are called pattern-recognition receptors (PRRs)[6,7]. The PRRs, localised on immune cells and in body fluid as soluble factors, possess a higher numerical variance than those of vertebrate organisms [8–10]. Among the most common PAMPs, there are components of the bacterial cell wall such as lipopolysaccharide (LPS), peptidoglycans (PGN) and lipopeptides, as well as flagellin, DNA and double-stranded RNA [11]. Molecular analysis of immune functions in the sea urchin reveals a very high degree of complexity through the presence of a complement system that appears to have multiple alternative pathways and diverse activators [1]. The immune system of the sea urchin also includes multiple sets of lectins, proteins with different antimicrobial activities, Toll-like receptors and associated signalling proteins [6]. It is probable, that there are yet more components yet to be described. Flow cytometry-based studies in PAMP-challenged *P*. *lividus* coelomocytes, identified increases in ROS production and the number of phagocytic cells [12]. However, little is known on the molecular mechanisms and the cellular processes that are activated, in this sea urchin, in response to the immune stimulation.

Based upon these considerations, we used a label free Mass spectrometry (Mass-spec) approach to identify differences in the abundance of proteins following bacterial LPS treatment and a bioinformatics approach to investigate the possible mechanisms and pathways modulated by these factors.

## Materials and methods

### Animals

A sample of 40 adult individuals of sea urchin (*P*. *lividus*), were collected from waters off Palermo, along the North coast of Sicily, at a depth of 5–10 m, near a meadow of *Posidonia oceanica* (commonly known as Neptune grass or Mediterranean tapeweed). The animals were maintained at 12–15°C, comparable to coastal temperatures, in an aerated aquarium with filtered sea water and a 10 h:14 h light:dark cycle. Seawater was prepared using Instant Ocean Sea Salt (Mentor, OH, USA) dissolved in deionised water corrected for salinity and pH. A small volume of water (10–20 L) was changed weekly, and the animals were fed once a week with commercial invertebrate food (Azoo, Taikong Corp., Taiwan). Sea urchins were acclimatised for at least 4 weeks, a time period deemed sufficient for immunological studies in the Mediterranean sea urchin *P*.*lividus* [12–15].

### Treatment of animals with LPS

**Fig 1:**
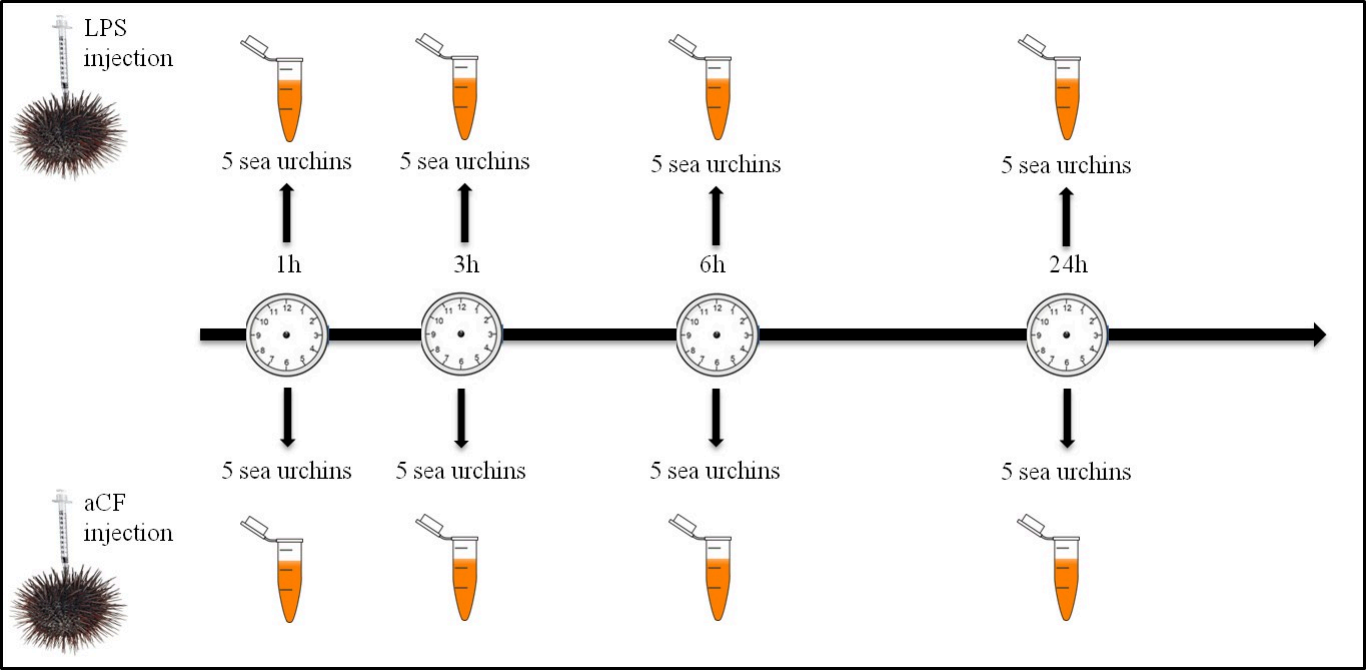
Graphical treatment scheme. A total of 40 animals were divided into eight groups of five animals. Four groups of sea urchins received an injection of 2**μ**g/mL of LPS (treated groups) and the remaining four groups received an injection of aCF (control groups). Coelomocytes from animals of treated and control groups were collected at 1, 3, 6 and 24h.

Different adult individuals of *P*. *lividus* received injections, into the coelomic cavity through the peristomial membrane, of 2 μg commercial lipopolysaccharide (LPS; *Escherichia coli;* Sigma-Aldrich cod. L-4524) *per* 1 mL of coelomic fluid. The reagent was resuspended in artificial coelomic fluid (aCF) (10 mM CaCl_2_; 14 mM KCl; 50 mM MgCl_2_; 398 mM NaCl; 1.7 mM Na_2_HCO_3_; 25 mM Na_2_SO_4_) as suggested by Terwilliger [16]. Control individuals were injected with 100μL of aCF. Subsequently, the coelomic fluid (4 mL) was withdrawn by syringe preloaded with isosmotic anticoagulant solution (ISO–EDTA; 0.5 M NaCl, 20 mM Tris-HCl, and 30 mM EDTA; pH 7.4), 1, 3, 6, 24 hours post LPS treatment (HPLT). Cells were washed with ISO-EDTA and counted using a Burker chamber. Coelomocytes from five animals (1 × 10^7^ cells), for each treated sample and relative control, were pooled together (Fig 1). After centrifugation (900 × g for 10 min at 4°C), the pellet containing the sea urchin immune cells was aliquoted at a density of 1 × 10^7^ cells mL^—1^.

### Extraction of protein from coelomocytes

Total coelomocyte counts (TCCs) were performed using a Burker chamber under a light microscope (Leica DMLB equipped with a digital camera Leica DC 200, Germany). The count values were calculated using the average numbers of coelomocytes observed in 30 microscopic fields for the examined animals.

The pellets containing the cells were lysed in RIPA buffer (20 mM Tris-HCl (pH 7.5) 150 mM NaCl, 1 mM Na_2_ EDTA 1 mM EGTA, 1% NP-40, 1% sodium deoxycholate 2.5 mM sodium pyrophosphate, 1 mM b-glycerophosphate, 1 mM Na_3_VO_4_, 1 μg/ml leupeptin) with anti-protease cocktail (Sigma) and were centrifuged at 10,000 g for 30 min at 4°C to remove any precipitate. Protein concentrations were determined by Quibit fluorimetry (Quibit 2.0 Fluorometer), and sample aliquots were stored at -80°C until use.

### Sample preparation for proteomics analysis

Lyophilised lysed cell samples were dissolved in 10 μL 0.5% RapiGest SF (Waters Corporation, Milford, MA) solution (1 mg RapiGest SF dissolved in 200 μL LC-MS water). As the samples contained RIPA buffer, solvent exchange was performed on 3 μL samples using 10 kDa Amicon Ultra centrifugal filters (Merck KGaA, Darmstadt, Germany). Samples were diluted to 200 μL with 200 mM ammonium bicarbonate and washed twice on the centrifugal filter. In the final step solvent was exchanged to 50 mM ammonium bicarbonate. Protein concentration was determined using a Nanodrop spectrophotometer and 25 μg aliquot of each sample was digested in solution. Subsequently 20 μL sample was denatured and alkylated in the presence of 5 μL 0.5% RapiGest and 2 μL 200 mM DTT at 60°C for 30 minutes. This was followed by alkylation using 2.5 μL 200 mM iodoacetamide and 5 μL 200 mM ammonium bicarbonate buffer in the dark at room temperature for 30 minutes. Next, Mass-spec grade Lys-C-tripsin mixture (Promega, Madison, WI) was added at 1:100 ratio and incubated at 37°C for 1 hour. This was followed by the addition of trypsin (Promega, Madison, WI) at 1:25 ratio and 2 hour incubation at 37°C. Digestion was quenched by the addition of formic acid (FA). Samples were dried and desalted using Pierce C18 spin columns (Thermo Fisher Scientific, Waltham, MA).

### Label free shotgun proteomics

Peptides were analysed using a Maxis II ETD Q-TOF (Bruker Daltonics, Bremen, Germany) coupled to an Ultimate 3000 nanoRSLC system (Dionex, Sunnyvale, CA, USA) via CaptiveSpray nanoBooster ion source. Samples were dissolved in 25 μl of 2% ACN (Acetonitrile), 0,1% FA and 4 μl was injected onto an Acclaim PepMap100 C-18 trap column (100 μm x 20 mm, Thermo Fisher Scientific, Waltham, MA). Trapping was performed with 0.1% TFA for 8 min with a flow rate of 5 μl/min. Separation of peptides was achieved on an Acclaim PepMap RSLC 75 μm x 50 cm analytical column (Thermo Fisher Scientific, Waltham, MA) at 48°C at a flow rate of 270 nL/min, using 0.1% FA as solvent A, and acetonitrile with 0.1% FA as solvent B. An initial gradient of 4% solvent B was utilised from 0 to 11 minutes, followed by a 90 minute gradient to 50% solvent B, with a further increase in concentration to 90% solvent B in 1 minute which was maintained for 5 minutes, followed by equilibration at 4% solvent B for 20 minutes. Blanks were injected between each sample to avoid carryover. Data dependent analysis was performed using fix cycle time of 2.5 sec. MS spectra were recorded in the *m/z* 150–2200 mass range at 3 Hz, while Collision-Induced Dissociation (CID) was performed on multiply charged precursors at 16 Hz for abundant ions and at 4 Hz for low abundance ions. Following each run the raw data were recalibrated for the internal sodium formate mass calibrant using the Compass DataAnalysis software 4.3 (Bruker Daltonics, Bremen, Germany). Proteins were identified using the in-house Mascot server v.2.5 (Matrix Science, London, UK) by searching against *Strongylocentrotus purpuratus* protein sequences (29720 entries) downloaded from Uniprot (www.uniprot.org). The following search parameters were set: trypsin enzyme, maximum of two missed cleavages, carbamidomethylation as fixed modification, methionine oxidation and deamidation (N,Q) as variable modifications. Precursor tolerance was set to 7 ppm, while the MS/MS tolerance was 0.05 Da. Label free quantification was performed on a sequence database, created using proteins identified from the Mascot search, employing the Andromeda search engine via MaxQuant software version 1.5.3.30 [17]. Proteins and peptides were accepted at 1% false discovery rate (FDR). The LC-MS/MS runs were aligned using the “match between runs” feature (match time window 0.8 min, alignment time window 15 min). MaxQuant LFQ algorithm was used and LFQ values were used to compare fold changes in protein abundances among control and treated samples. The mass spectrometry proteomics data have been deposited to the ProteomeXchange Consortium [18] via the PRIDE [19] partner repository with the dataset identifier PXD008439.

### Bioinformatics analysis

Proteins identified by Mass Spectrometry were examined using Panther (Protein Analysis through Evolutionary Relationship, Version 13.1) [20], a classification system for proteins and their genes. Proteins found to be up- or down-modulated relative to the control (log_2_-fold change <-0.5 or log_2_-fold change >0.5) were selected for further analysis. Protein-protein interaction networks were visualised using the “Search Tool for Recurring Instances of Neighbouring Genes” (STRING) [21–23]. The minimum required interaction score was set to 0.7 and disconnected nodes were hidden. Because many nodes were referred to by the LOC IDs, to simplify the readability of the figures, such identifiers were replaced with the Gene name or Gene symbol. The conversion table between STRING and UNIPROT identifiers is shown in S2 Table.

Pathway enrichment analysis was then performed using “Kyoto Encyclopedia of Genes and Genomes” (KEGG) [24–26] to determine the pathways in which the significantly up- and down-expressed proteins were implicated.

## Results

### Mass spectrometry analysis

**Fig 2:**
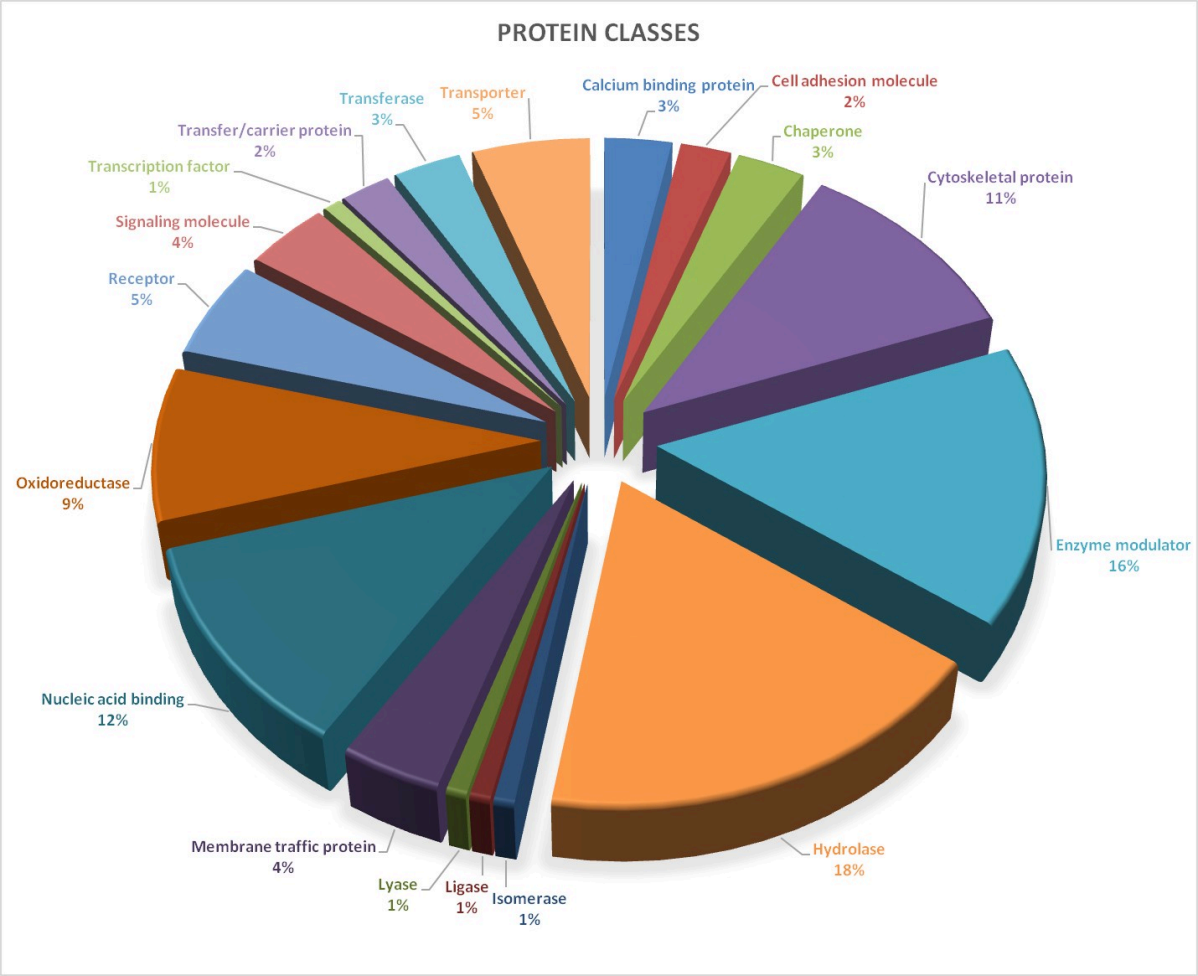
Protein classes identified by mass spectrometry. Proteins identified by Mass Spectrometry were examined using Panther (Protein Analysis through Evolutionary Relationship, Version 13.1). A total of 137 proteins was recognised and divided into eighteen classes: calcium binding protein, cell adhesion molecule, chaperone, cytoskeletal protein, enzyme modulator, hydrolase, isomerase, ligase, lyase, membrane traffic protein, nucleic acid binding, oxidoreductase, receptor, signaling molecule, transcription factor, transfer/carrier protein, transferase, transporter.

Proteins extracted from coelomocytes isolated from LPS treated sea urchins, were subjected to shotgun mass spectrometry analysis. The samples were analysed by the commercially available MaxQuant software [17] to obtain label free quantitative data. Altogether, 146 proteins, recognised by at least two unique peptides and 1% FDR, were identified and the relative abundance was estimated comparing data from treated samples with their controls. Furthermore, a first bioinformatics analysis, through the Panther Classification System tool, was performed. A total of 137 proteins was recognised (S1 Table) by the tool and divided into eighteen classes (Fig 2).

Cytoskeletal proteins, enzyme modulators (such as small GTPases, heterotrimeric G-proteins, protease inhibitors), hydrolases and nucleic acid binding factors were the most abundant classes (>10%) comprising, in total, 55.8% of the analysed proteins. In particular, cytoskeletal protein class was constituted of actin family (69.2%) and microtubule family cytoskeletal proteins (30.8%); enzyme modulator class was mainly represented by G-proteins (72.2%), G-protein modulators (11.1%), that could represent an important group of signaling proteins involved in immune function [1], and protease inhibitors (16.7%); hydrolase class was composed by proteases (88.9%) and deaminase (11.1%); nucleic acid binding classes were mainly represented by DNA (53.3%) and RNA (46.7%) binding proteins.

Subsequently, based on the MaxQuant label free quantitative results, the 88 most abundant proteins, each identified by at least two unique peptides, were used for further bioinformatics analysis. Analysis of the data indicates that the highest changes occurred after 3 hours after the LPS treatment, suggesting a very fast response to treatments. Some of the data from mass spectrometry was verified by a different technique. In particular, the protein expression of HSP70 (UniProt ID: W4Y0E3) and β-Thymosin (UniProt ID: W4ZCZ4) were verified by western blotting and dot-blot respectively. These data were already published as part of a study focused on the immune response to bacterial LPS in the *Paracentrotus lividus* [27].

Therefore, the mass spectrometry analysis identified proteins and protein classes involved and differently modulated in the immune *P*. *lividus* response to LPS challenge.

### Physical and functional association of proteins

The IDs of proteins that were modulated (Log_2_ Fold-change >0.5 and <-0.5), after each treatment, compared to their controls, were submitted to the STRING database for the investigation of known and predicted protein-protein interactions.

**Fig 3:**
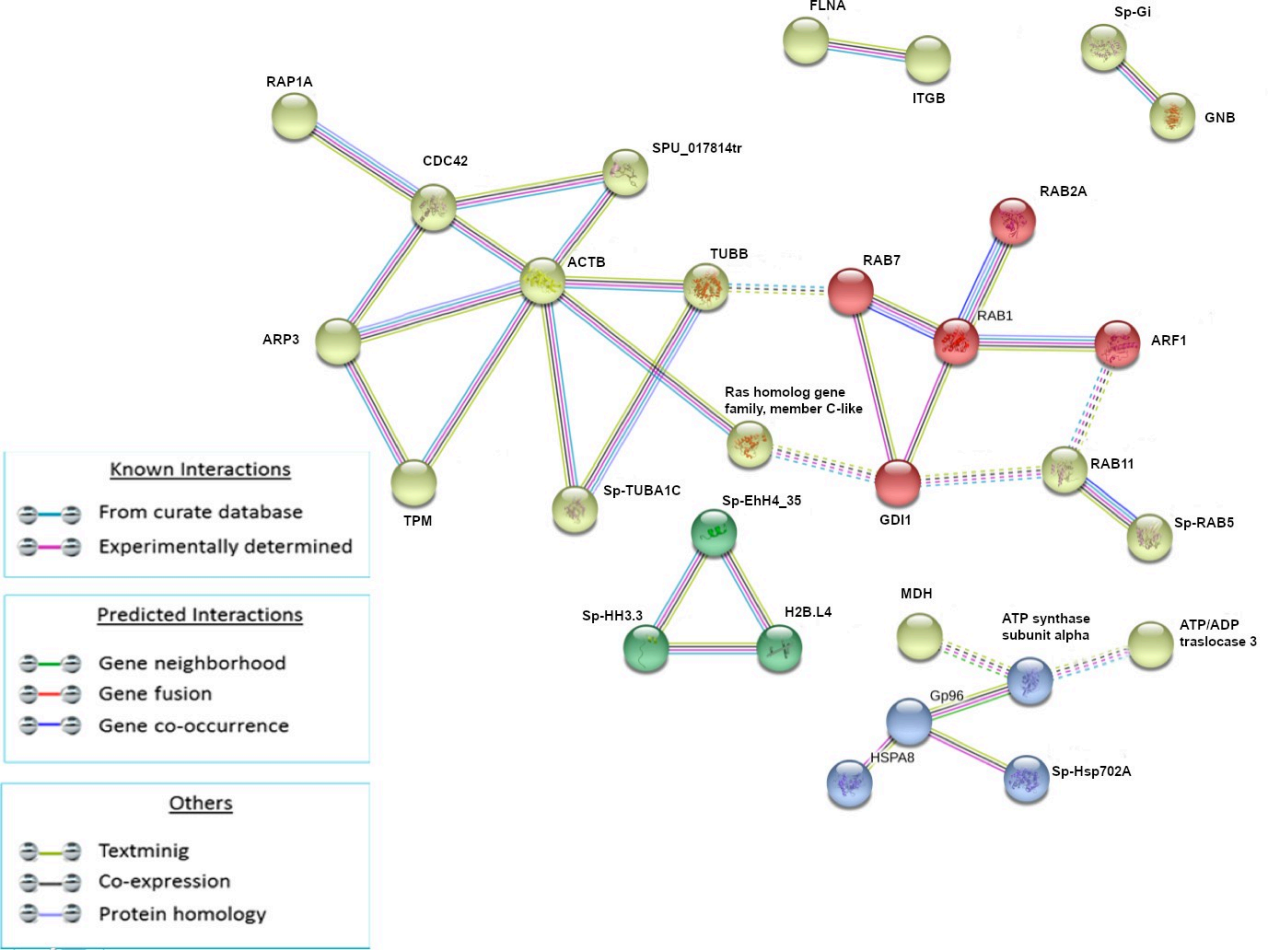
Analysis of known and predicted protein-protein interaction networks at 1 HPLT. Modulated proteins (Fold change >0.5 and <-0.5) were used as input for STRING analysis to reveal known and predicted protein-protein interaction networks. Proteins are indicated by nodes labeled with the encoding gene symbol. Additional cluster analysis was used to colour the nodes of the interaction networks (k-means = 3). The analysis showed the modulation of a network of interaction that was mainly composed of cytoskeleton and cytoskeleton-related factors (yellow cluster), a network mainly composed of RAS superfamily GTPase members (red cluster), a network mainly composed of heat shock proteins (blue cluster) and a network composed of the histone proteins (green network).

We selected the k-means unsupervised clustering algorithm based on adjacent matrix, which groups molecules based on pre-specified criteria. In particular, after 1HPLT, the protein networks were divided into four clusters for total modulated proteins because this value is the best to show non-overlapping clusters (Fig 3).

Following LPS treatment (1HPLT), four groups of protein-protein interactions were present among proteins that resulted significantly up and down modulated. The yellow network (ACTB, SP-TUBA1C, TUBB, SPU_017814tr, CDC42, RAP1A, ARP3, TPM, Ras homolog gene family, member C-like), whose hub is the ACTB, was mainly composed of cytoskeleton and cytoskeleton-related factors; the red network (RAB1, RAB2A, RAB7, GDI1, ARF1), whose hub is RAB1, was mainly composed of RAS superfamily GTPase members. The two clusters were linked primarily by interactions between TUBB and Ras homolog gene family, member C-like of yellow network and, respectively, RAB7 and GDI1 of the red network. The blue network (HSPA8, Gp96, Sp-HSP702A, ATP synthase subunit alpha) was mainly composed of heat shock proteins and the green network was composed of histones Sp-EhH4_35, Sp-HH3.3 and H2B.L4.

**Fig 4:**
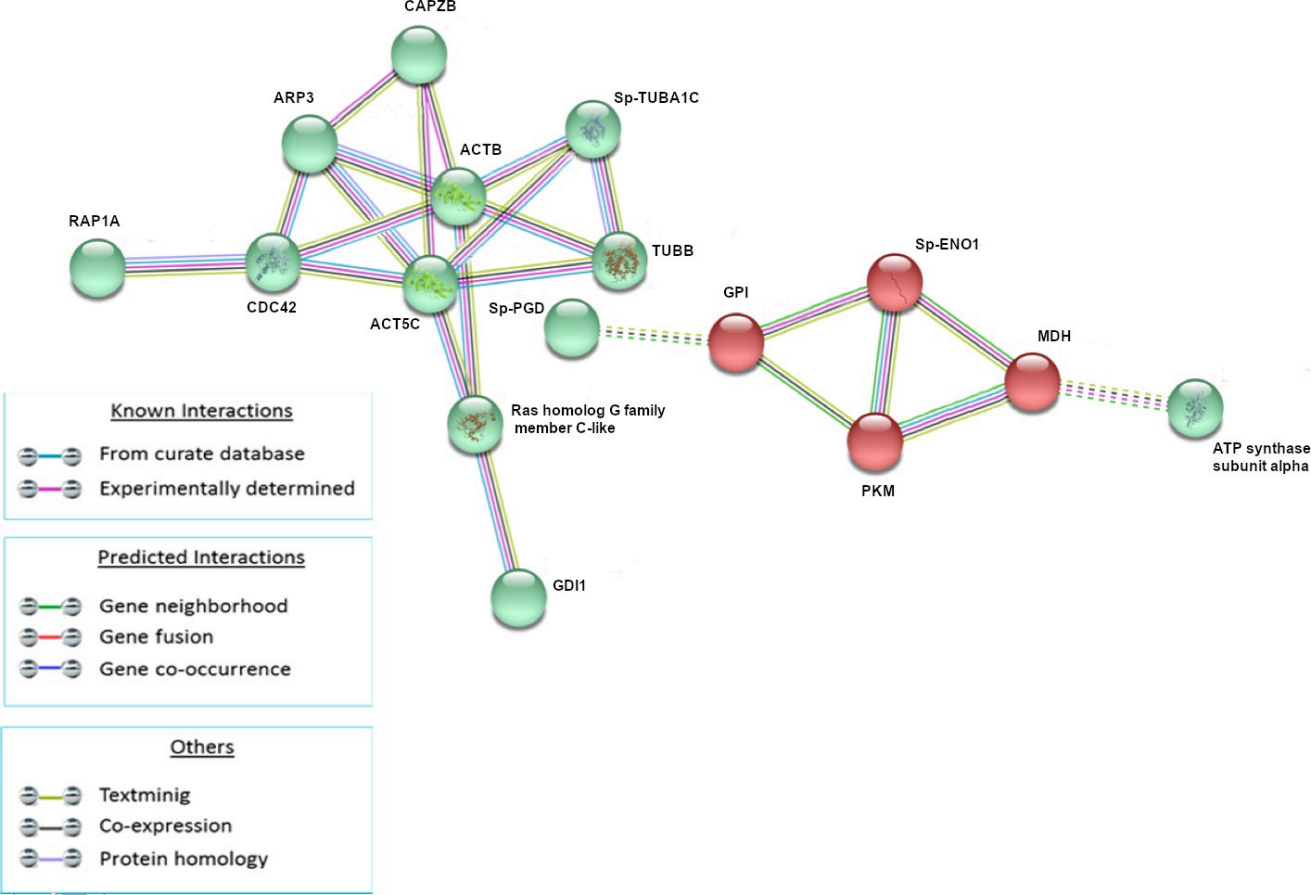
Analysis of known and predicted protein-protein interaction networks at 3 HPLT. Modulated proteins (Fold change >0.5 and <-0.5) were used as input for STRING analysis to reveal known and predicted protein-protein interaction networks. Proteins are indicated by nodes labeled with the encoding gene symbol. Additional cluster analysis was used to colour the nodes of the interaction networks (k-means = 2). The analysis showed the modulation of a network of interaction that was mainly composed of proteins involved in cytoskeleton and RAS signaling (green cluster) and of a second network mainly composed of proteins involved in energetic homeostasis (red cluster).

The analysis of modulated proteins after 3h of treatment showed the presence of two main clusters (Fig 4). The green cluster (RAP1A, CDC42, ACT5C, ARP3, CAPZB, Sp-TUBA1C, ACTB, TUBB, Ras homolog gene family, member C-like, GDI1) is mainly composed of protein involved in cytoskeleton and RAS signaling while the red cluster (Sp-ENO1, GPI, PKM, MDH) is mainly composed of proteins involved in energetic homeostasis.

**Fig 5:**
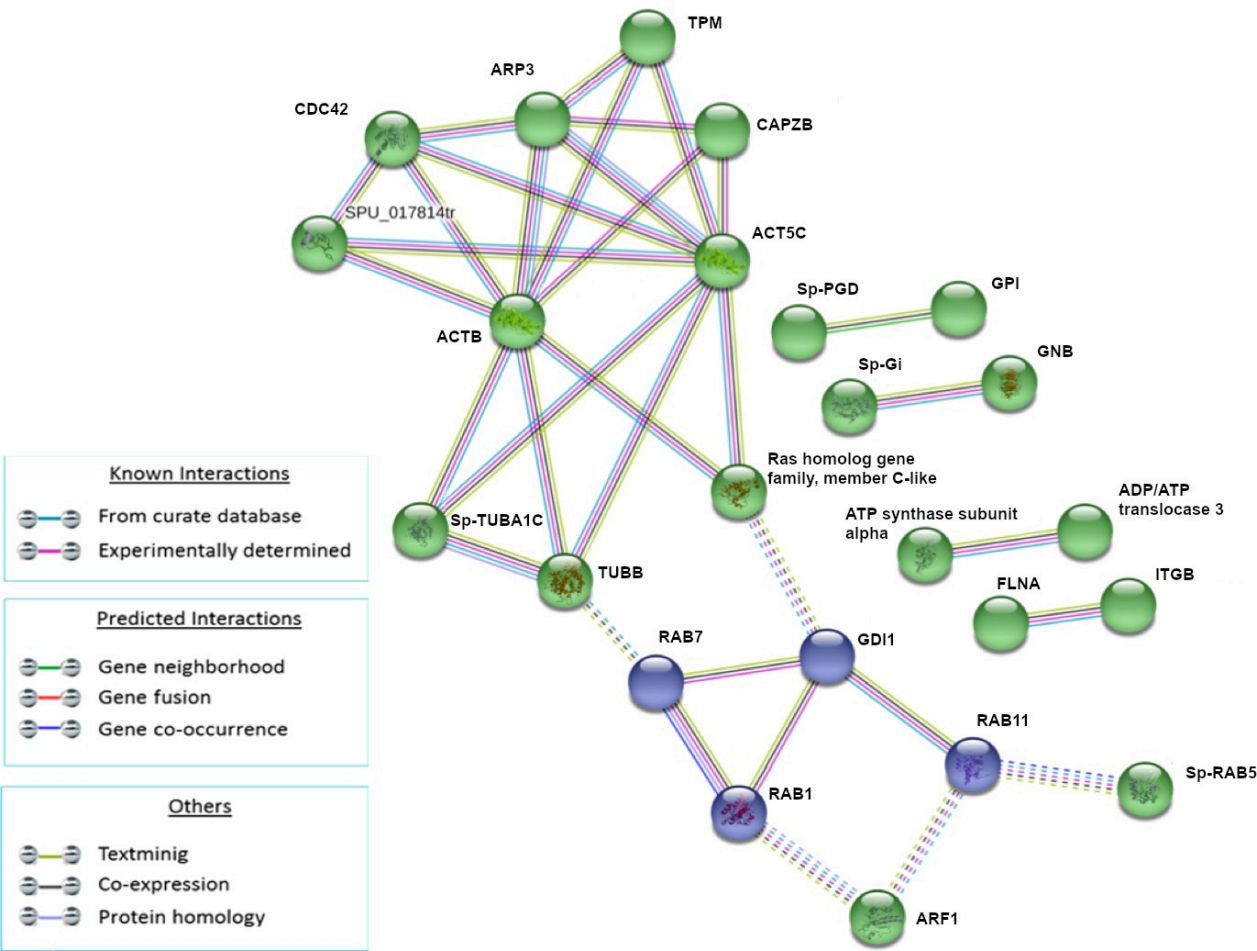
Analysis of known and predicted protein-protein interaction networks at 6 HPLT. Modulated proteins (Fold change >0.5 and <-0.5) were used as input for STRING analysis to reveal known and predicted protein-protein interaction networks. Proteins are indicated by nodes labeled with the encoding gene symbol. Additional cluster analysis was used to colour the nodes of the interaction networks (k-means = 2). The analysis showed the modulation of a network of interaction that was mainly composed of proteins involved in the cytoskeleton organization and RAS GTPases (green cluster), while the latter was composed of RAB proteins specifically involved in vesicles and endosome transport, fusion and recycling.

After 6h of treatment, the analysis showed two clusters represented by the green and violet groups (Fig 5). The former (TPM, CDC42, CAPZB, SPU_017814tr, ACT5C, ACTB, Sp-TUBA1C, TUBB, Ras homolog gene family, member C-like) was composed of proteins related to cytoskeleton organization and RAS GTPases, while the latter was composed of RAB proteins specifically involved in vesicles and endosome transport, fusion and recycling.

**Fig 6:**
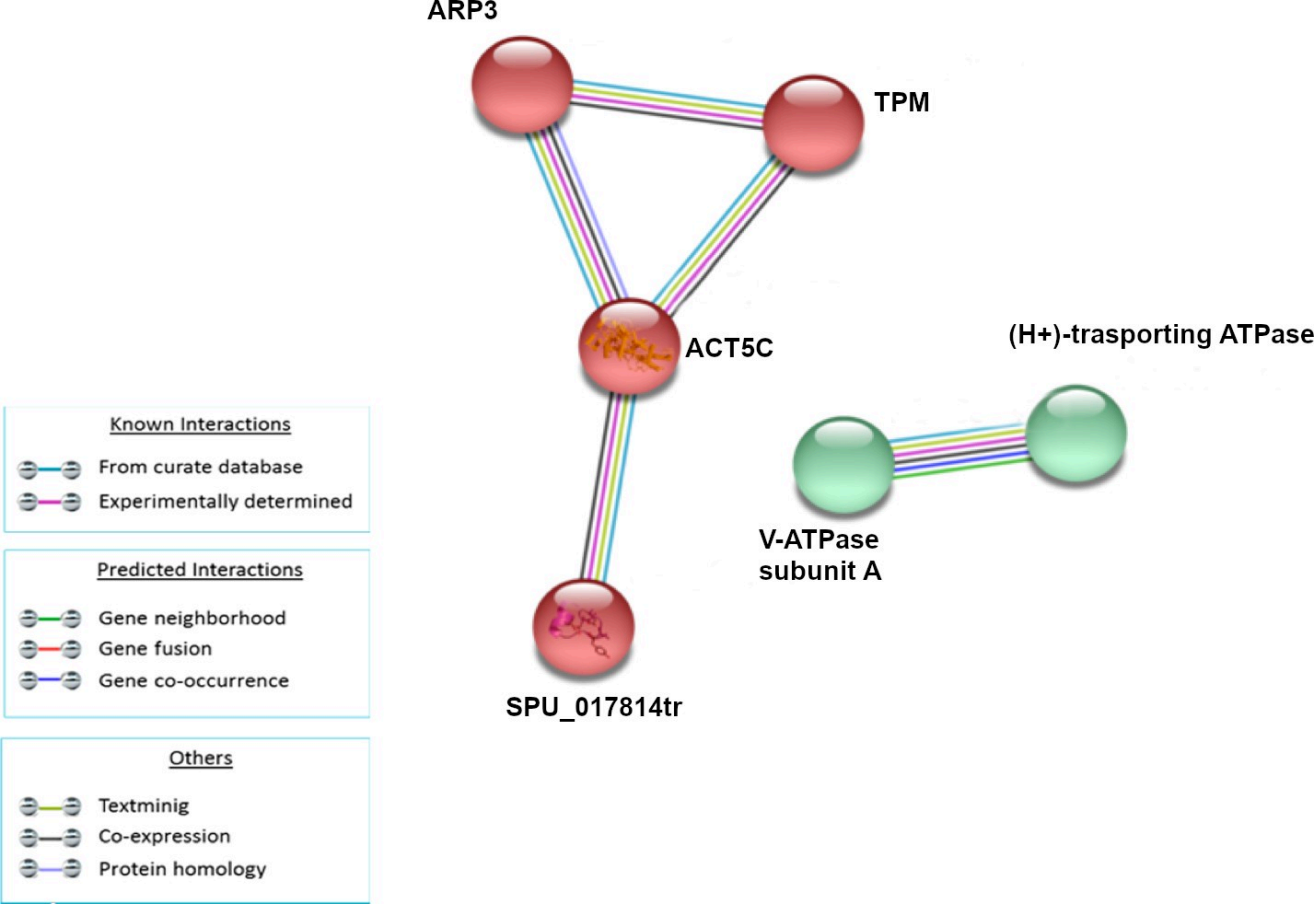
Analysis of known and predicted protein-protein interaction networks at 24 HPLT. Modulated proteins (Fold change >0.5 and <-0.5) were used as input for STRING analysis to reveal known and predicted protein-protein interaction networks. Proteins are indicated by nodes labeled with the encoding gene symbol. Additional cluster analysis was used to colour the nodes of the interaction networks (k-means = 2). The analysis showed the modulation of a network of interaction that was mainly composed of proteins related to cellular cytoskeleton (red cluster).

The analysis of modulated proteins after 24h of treatment showed a single small cluster (Fig 6) composed of proteins related to cellular cytoskeleton (ARP3, TPM, ACT5C, SPU_017814tr).

This result seemed to suggest that cytoskeleton reorganisation could be the last process to be restored after LPS treatment.

The STRING tool has clearly shown, through the analysis of the sea urchin coelomocytes proteome, the presence of known and predicted protein-protein interaction networks that are differently modulated by LPS at different time points.

### Cell pathways modulated by LPS treatment

**Fig 7:**
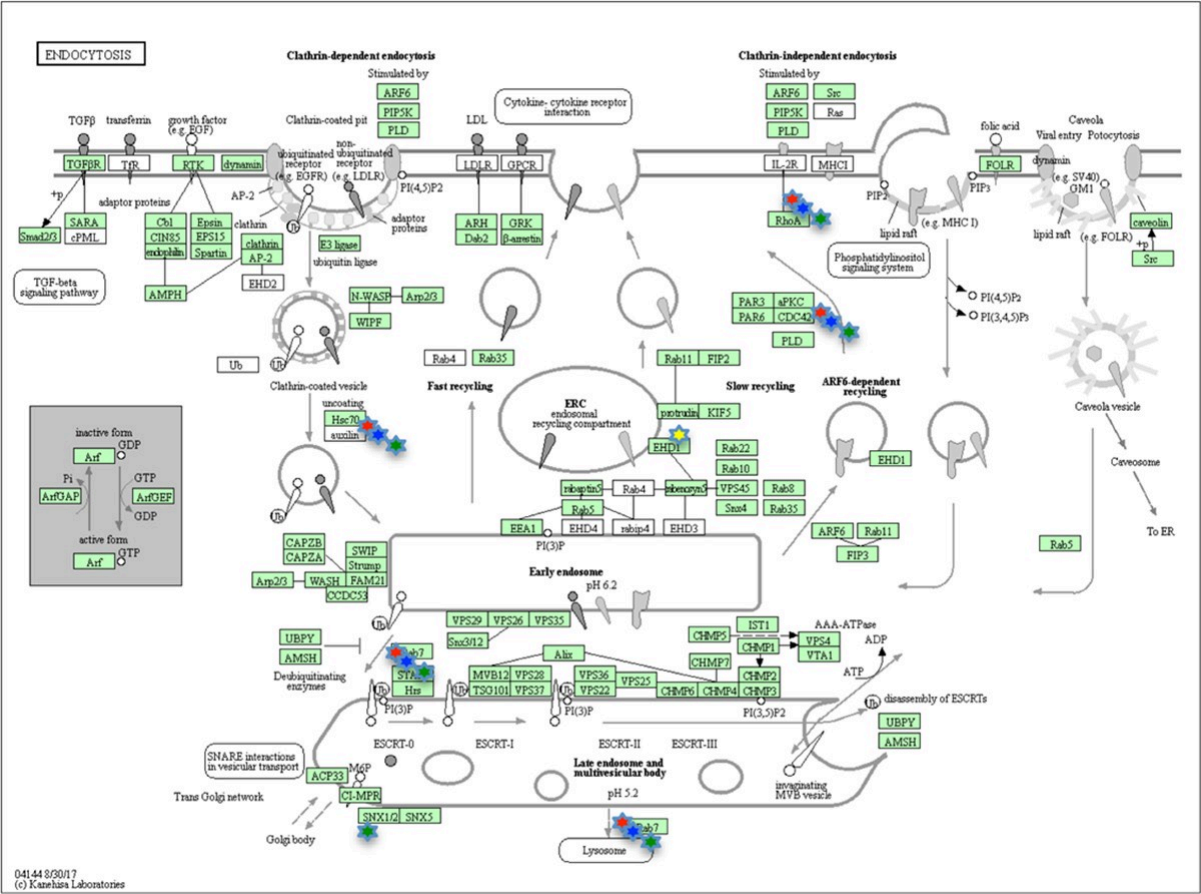
KEGG Endocytosis pathway. Endocytosis pathway as showed by the KEGG database. Proteins in the pathway are depicted by boxes while arrows depict signaling routes. Red stars correspond to modulated proteins, identified by the MS analysis, at 1HPLT (Hours post LPS treatment), blue stars at 3 HPLT, green stars at 6HPLT and yellow stars at 24HPLT.

**Fig 8:**
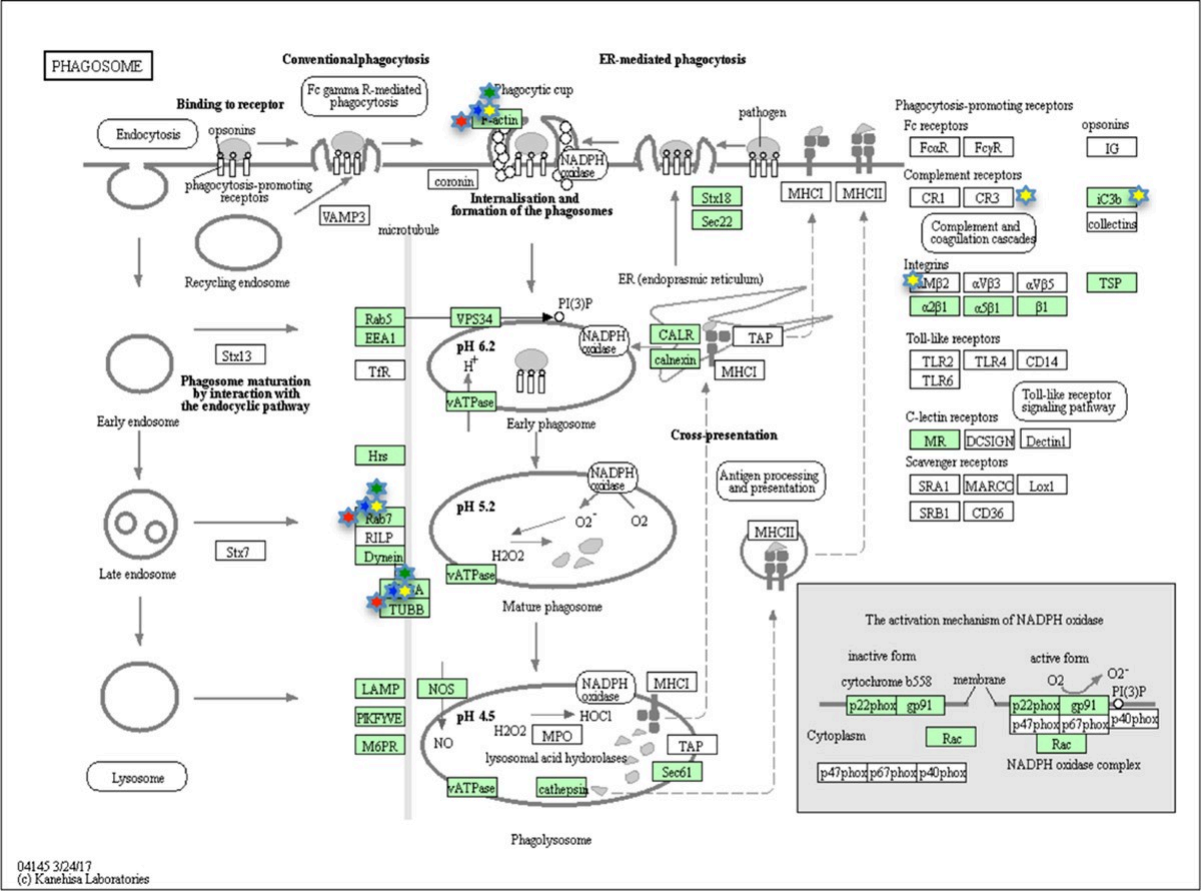
KEGG Phagosome pathway. Phagosome pathway as showed by the KEGG database. Proteins in the pathway are depicted by boxes while arrows depict signaling routes. Red stars correspond to modulated proteins, identified by the MS analysis, at 1HPLT ((Hours post LPS treatment), blue stars at 3 HPLT, green stars at 6HPLT and yellow stars at 24HPLT.

To investigate which pathways could be modulated by the LPS treatment, at each time step, the differently expressed proteins were submitted to the KEGG database. The analysis showed at 1, 3 and 6HPLT, the statistically significant (False Discovery Rate < 0.05) modulation of the Endocytosis (Fig 7) and Phagosome (Fig 8) pathways. At 24HPLT, only the phagocytosis pathway was significantly modulated.

This result led to the identification of the Endocytosis and Phagosome as the two main pathways involved in the *P*. *lividus* immune response to LPS treatment.

## Discussion

Sea urchin immune system, and in particular that of the Mediterranean *Paracentrotus lividus*, is a complex defence system able to protect the animal from pathogen attacks. These animals possess an “innate” immune system that acts through cellular and humoural responses [1,4]. The cellular response is mediated by a particular class of cells, the coelomocytes, that circulate in the coelomic cavity and can infiltrate tissues and organs being the first mediators of allograft rejections; they act, through different activating pathways [4,6,15,28,29] in response to host invasion, injury and cytotoxic agents. The humoral response is mainly due to molecules like lectins, cytokines and profilins, to the complement system and antimicrobial peptides [30–32]. For the purpose of this study, we treated *Paracentrotus lividus* sea urchins with LPS, at different exposure times, to induce an immunological response, and then, through a label free mass spectrometry approach, we analyzed the protein expression pattern to identify mechanisms, processes and pathways involved in the immune response.

Due to the unavailability of the complete sequenced genome of the *P*. *lividus* we have used a validated cross species approach [33–39] utilising data from a very phylogenetically close echinoderm species, the *S*. *purpuratus* sea urchin.

In 2013, Dheilly et al. [40] published a study in which they used shotgun mass spectrometry to describe a number of proteins with possible immune function in the purple sea urchin. In particular, they performed a proteomic analysis of the coelomic fluid of the purple sea urchin providing an important overview of the proteins expressed in this echinoderm. In our research, the proteomic analysis was performed on the coelomocytes, the immune cells of the sea urchin *P*. *lividus*, to reveal, over time, processes, clusters of interaction and pathways that are differently modulated in response to the LPS treatment. This approach was independent of the subpopulation changes, deeply analyzed elsewhere [27], that occurred in response to the immunological stimulus and of the causes of protein modulation that could be due to *de novo* synthesis, degradation and translation impairment in a cell population or to the contribution of migrating cells, or both. Furthermore, not all the proteins expressed during the immune response were identified by our experiments. In fact, some proteins are mainly secreted and present in the coelomic fluid, others could be more difficult to extract due to their solubility properties.

In our study, STRING analysis of the modulated proteins following LPS treatment for 1 hour, resulted in four main clusters of protein-protein interactions. The biggest, in terms of protein nodes, was constituted of cytoskeletal factors such as actins and β-tubulins, calmodulin, that controls the contractile activity of cytoskeleton [41], CDC42 and Ras homolog family members that control cytoskeleton dynamics. Previous studies of *Strongylocentrotus purpuratus* have shown the up-regulation of the transcription of cytoskeletal genes and cytoskeleton-regulating protein genes [1,42] at 24HPLT. To our knowledge our experiment is the first to monitor the expression of these factors in the *P*. *lividus* sea urchin, which represent the responsive proteins at 1, 3, 6 and 24h of treatment.

The strategic role of the cytoskeleton in innate immunity and cellular self-defense has been highlighted in other cell systems [43]. In fact, it was seen that the Nod1 protein could form multiprotein complexes with HSP90, the GTPases RAC1 or CDC42 and their bacterial GEF SopE, which in turn could induce actin-dependent membrane ruffling during invasion. Therefore, SopE-induced changes to RAC1 and CDC42 were detected by Nod1, which acted as a guardian of the cytoskeleton during *Salmonella enterica* infection [43].

RAC and CDC42 are responsible for the formation of morphologically different protrusions at the plasma membrane, such as lamellipodia and filopodia, but they both superintend peripheral actin polymerisation through the ARP2/3 complex, another complex that is modulated in our research. The ARP2/3 complex is a heptameric, actin-nucleation machine, and is present in all eukaryotic cells. It associates with the sides and the ends of existing actin scaffolds to form new branched filaments [44]. GTPases, RAC and CDC42, activate ARP2/3 indirectly through the Wiskott-Aldrich syndrome protein (Wasp) family members. *In vitro*, CDC42-GTP binds directly to Wasp, to relieve an intra or trans-molecular inhibitory interaction and expose a C-terminal ARP2/3 binding/activation site [45–47].

The protein RAP1 is a small cytosolic GTPase, that belongs to Ras-related protein family. It is quickly and transiently activated in response to chemo attractant signals and helps determine cell polarity by modulating cytoskeletons. The mechanisms by which RAP1 controls actin cytoskeletal reorganization in *Dictyostelium* have been reported, and RAP1 interacts with RAC-GEF1 *in vitro* and stimulates F-actin polymerization [48]. RAC family proteins are important regulators in actin cytoskeletal reorganization. In fact, *in vitro* binding assay using truncated RAC-GEF1 proteins demonstrated that RAP1 interacts with the RAC-GEF1.

GTPases of the RHO family can regulate cytoskeletal dynamics through multiple cellular functions including cell motility and polarity [49–51]. Their function is well regulated in space and time. Most RHO GTPases cycle between an inactive form characterised by the binding with the nucleotide GDP, and an active form characterised by the binding with the nucleotide GTP. The RHO family has at least twenty-two members in humans, grouped into eight subfamilies. The RHO subfamily includes the isoforms RHOA, RHOB, and RHOC, which share a high degree of homology, (84%), with most of the differences concentrated close to the C terminus [52]. RHOA, RHOB, and RHOC can all induce stress fibres when over-expressed. In fact, the *Clostridium botulinum* exoenzyme C3 transferase, is able to induce loss of stress fibres and inhibition of cell migration.

In our research, actin, tubulin RAP1A, CDC42, and RHOC were all modulated and part of a cluster of protein-protein interaction following LPS treatment. This evidence seems to suggest that LPS is able to induce an actin reorganisation that includes the modulation of the expression of the cytoskeleton proteins and the regulation of the factors that control the dynamics of the cytoskeleton. In addition, even if this study is independent on the specific cell type involved in the immune response, it is important to highlight that most of the coelomocytes in echinoids are of the large phagocytes class (up to 80% in *P*. *lividus*), characterised and described by an important and complex cytoskeletal organization [53,54]. The size of the cluster was similar from 1HPLT to 6HPLT and at 24HPLT it was clearly reduced. This could mean that the cytoskeleton perturbation induced by LPS begins immediately after the bacterial molecule injection, as showed at 1HPLT, and is maintained until 6HPLT when it starts to decrease, as showed at 24HPLT, where the only factors of the cluster were actins and calmodulin.

Another cluster of protein-protein interaction found modulated at 1HPLT constituted of RAB proteins. RAB GTPases represent a big family of small GTPases that are fundamental regulators of intracellular membrane traffic [55]. Different RAB GTPases localise to distinct membrane structures in order to control the specificity and directionality of pathways related to membrane trafficking, such as vesicular transport. The presence of specific and numerous intracellular compartments indeed requires a high order of transport regulation, controlled by RAB proteins through the microtubule and actin networks of motor protein-driven transport [56]. In our experiments, the presence of the red cluster at 1HPLT (Fig 3) and the violet cluster at 6HPLT (Fig 5), comprising different RABs and Rab-associated proteins, in association with the cluster of cytoskeleton proteins, suggested how LPS was able to induce the modulation of the intracellular membrane trafficking pathways. These data were supported and bioinformatically confirmed by KEGG analysis of the pathways modulated by the LPS treatment that identified Endocytosis (at 1,3 and 6HPLT) and Phagocytosis (from 1 to 24HPLT) as the two main perturbed pathways. Furthermore, the actin polymerisation is central to immune processes like phagocytosis and macro-pinocytosis, driving plasma membrane extensions that load external cargo and internalise them through the Endo- and Phagocytic processes.

Mass spectrometry analysis at 1HPLT, has shown the modulation of a cluster of protein-protein interaction constituted mainly of histone proteins (Fig 3). Histones are fundamental constituents of the Eukaryotic chromatin. Their primary function is linked to chromatin structure and transcriptional regulation. The specific role of histone proteins in innate immunity has been poorly investigated. It was seen that in a number of aquatic invertebrates, as well as in many other invertebrate and vertebrate species, that RNAs transcribing for core histones are up-regulated following immune stimuli or exposure to environmental stressors. Histones displayed antimicrobial properties against bacteria and parasites with the capacity to bind bacterial LPS and other pathogen associated molecules [57].

In parallel with the cytoskeleton reorganisation and the initiation of the endocytic and phagocytic processes that lead to the digestion of pathogen organisms, bacterial LPS can also initiate protective strategies in which the histone proteins can act as antimicrobial proteins. The establishment of a stress process also assists this defense mechanism [58–64]. Indeed, our study has shown the activation of stress processes in the Mediterranean *P*. *lividus* sea urchin, at 1HPLT, through the presence of a protein-protein cluster basically constituted of HSP proteins (Fig 3). All these expensive events are supported by the burst of energy represented, at 3HPLT, released by proteins (red cluster) related to energetic homeostasis (Fig 4). In fact, processes such as phagocytosis, which requires actin polymerization, vacuole maturation and acidification, and antimicrobial proteins production, require a large amounts of energy [65–67]. Based on our results we have shown that this is provided by the up-modulation of proteins like glucose-6-phosphate isomerase, pyruvate kinase, malate dehydrogenase and enolase.

**Fig 9:**
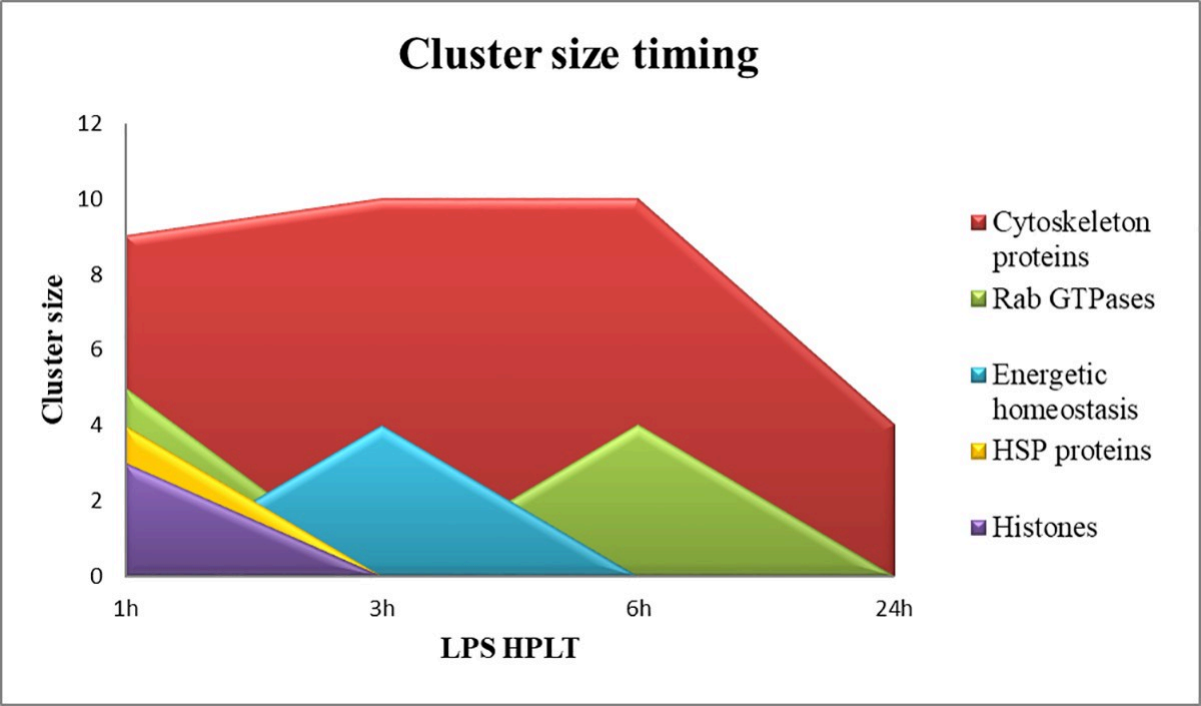
Modulation of the size of clusters during the LPS treatment. Area chart representing the timing profile of cluster sizes in terms of number of STRING nodes, at 1, 3, 6 and 24HPLT for Cytoskeleton proteins (red), RAB GTPases (lime), Energetic homeostasis (light blue), HSP proteins (yellow), Histones (purple).

The timing of the size of the modulated clusters showed that cytoskeleton reorganisation, RAB proteins, HSP protein and histone protein groups were mainly inflected as soon as LPS was injected into the animals (Fig 9).

As previously stated the processes associated with these protein groups are costly in terms of energy consumption. Thus cells are required to increase the ATP production as soon as their reserves decrease and this could explain why the energetic cluster size reached its maximum amplitude 3 hours post LPS treatment. Furthermore, at 24HPLT, the trend shows that the cell system is restored to the control because the sizes of the clusters and the modulated pathways are reduced, as shown by the KEGG pathway analysis (Fig 7, Fig 8).

The present study demonstrates, through a label free shotgun mass spectrometry approach, for the first time, to our knowledge, in the Mediterranean sea urchin *P*. *lividus*, the concomitant modulation of different processes and mechanisms that are essential to the understanding of the immunological response to pathogen attacks and, more in general, the immune system of this animal.

## Conclusions

Our results represent an important step in the study and research of the immune mechanisms of the Mediterranean sea urchin *Paracentrotus lividus*. Using label free mass spectrometry, we have identified a number of proteins that are differently modulated following LPS injection at 1, 3, 6 and 24 hours after treatment. Analysing these results we have identified protein clusters by STRING analysis and protein pathways based on the KEGG database, which are affected by bacterial LPS treatment. In particular we have shown how the bacterial wall constituent is sufficient to set off an immune response inducing cytoskeleton reorganization, the appearance of clusters of HSP and histone proteins and the activation of the endocytosis and phagocytosis pathways. This pilot study represents a new important step in the comprehension and knowledge of the *P*. *lividus* sea urchin immune system. Further experiments in order to determine biological variability, and the response in individual organisms and species are planned in the future.

## Supporting information

S1 Table PANTHER analysis table. Panther classification of 137 identified proteins by the bioinformatic tool.
(DOCX)

S2 Table UNIPROT-STRING conversion table. Conversion table between UNIPROT and STRING identifiers. Where possible, protein name, gene name, gene symbol and ortholog have been added.
(DOCX)
