# Supplementary material for: Changes in the proteome of sea urchin Paracentrotus lividus coelomocytes in response to LPS injection into the body cavity
Source: PLoS One. 2020 Feb 19;15(2):e0228893. doi: 10.1371/journal.pone.0228893 (PMC7030939; doi:10.1371/journal.pone.0228893)
Supplement: S1 Table — Panther classification of 137 identified proteins by the bioinformatic tool. (DOCX) [file pone.0228893.s001.docx]

**S1 Table**

| **GENE ID** | **MAPPED Ids** | **Gene name; Gene symbol; Otholog** | **Panther family/subfamily** | **Panther protein class** | **Panther GO-Slim Molecular Function** | **Panther GO-Slim Biological Process** | **Panther GO-Slim Cellular Compnent** |
| --- | --- | --- | --- | --- | --- | --- | --- |
|  |  |  |  |  |  |  |  |
| STRPU\|EnsemblGenome=SPU_024622\|UniProtKB=W4Z8Z8 | W4Z8Z8 | Uncharacterized protein; unassigned; ortholog | 10-FORMYLTETRAHYDROFOLATE DEHYDROGENASE (PTHR11699:SF190) | dehydrogenase(PC00092) | oxidoreductase activity; | - | - |
| STRPU\|EnsemblGenome=SPU_018866\|UniProtKB=W4YSS9 | W4YSS9 | 40S ribosomal protein S8; unassigned; ortholog | 40S RIBOSOMAL PROTEIN S8 (PTHR10394:SF3) |  | structural constituent of ribosome; | cellular component biogenesis; cellular process; nitrogen compound metabolic process; rRNA metabolic process | cytosol; organelle; ribosome |
| STRPU\|EnsemblGenome=SPU_026944\|UniProtKB=W4ZFJ5 | W4ZFJ5 | Uncharacterized protein; unassigned; ortholog | ADP-RIBOSYLATION FACTOR 1 (PTHR11711:SF308) |  | - | cellular process; intracellular protein transport; protein localization; vesicle-mediated transport | plasma membrane |
| STRPU\|EnsemblGenome=SPU_013662\|UniProtKB=W4YD34 | W4YD34 | Uncharacterized protein; unassigned; ortholog | RIBOSOMAL PROTEIN S3 (PTHR11760:SF9) | ribosomal protein(PC00202) | DNA N-glycosylase activity; damaged DNA binding; lyase activity; structural constituent of ribosome | DNA repair; cellular process; nitrogen compound metabolic process; regulation of nucleobase-containing compound metabolic process; response to stress | cytosol; nucleus; ribosome |
| STRPU\|EnsemblGenome=SPU_022476\|UniProtKB=W4Z2Y1 | W4Z2Y1 | Transaldolase; unassigned ortholog | TRANSALDOLASE (PTHR10683:SF18) |  | - | - | - |
| STRPU\|Gene=COII\|UniProtKB=P15545 | P15545 | Cytochrome c oxidase subunit 2; COII ortholog | CYTOCHROME C OXIDASE SUBUNIT 2 (PTHR22888:SF9) | oxidoreductase(PC00176) | cation transmembrane transporter activity; hydrogen ion transmembrane transporter activity; oxidoreductase activity | biosynthetic process; cellular process; nitrogen compound metabolic process; nucleobase-containing compound metabolic process; oxidative phosphorylation; phosphate-containing compound metabolic process; respiratory electron transport chain | - |
| STRPU\|EnsemblGenome=SPU_009481\|UniProtKB=A0A0C3SG04 | A0A0C3SG04 | Uncharacterized protein; unassigned ortholog | ACTIN, CYTOPLASMIC 1-RELATED (PTHR11937:SF155) | actin and actin related protein(PC00039) | structural constituent of cytoskeleton | cellular component organization; cytokinesis; endocytosis; exocytosis; intracellular protein transport; mitosis | actin cytoskeleton; intracellular |
| STRPU\|EnsemblGenome=SPU_018717\|UniProtKB=W4YSD0 | W4YSD0 | Uncharacterized protein; unassigned ortholog | ACTIN-RELATED PROTEIN 2 (PTHR11937:SF37) | actin and actin related protein(PC00039) | structural constituent of cytoskeleton | cellular component organization; cytokinesis; endocytosis; exocytosis; intracellular protein transport; mitosis | actin cytoskeleton; intracellular |
| STRPU\|EnsemblGenome=SPU_002374\|UniProtKB=W4XG61 | W4XG61 | Uncharacterized protein; unassigned ortholog | FERMITIN 2-RELATED (PTHR16160:SF13) |  | - | - | - |
| STRPU\|EnsemblGenome=SPU_013770\|UniProtKB=W4YDE0 | W4YDE0 | Adenosylhomocysteinase; unassigned ortholog | ADENOSYLHOMOCYSTEINASE (PTHR23420:SF14) | hydrolase(PC00121) | hydrolase activity | cellular amino acid metabolic process; cellular process; coenzyme metabolic process; nitrogen compound metabolic process; nucleobase-containing compound metabolic process; sulfur compound metabolic process | cytosol |
| STRPU\|EnsemblGenome=SPU_003825\|UniProtKB=W4XK99 | W4XK99 | Uncharacterized protein; unassigned ortholog | 14-3-3 PROTEIN EPSILON (PTHR18860:SF17) |  | - | - | - |
| STRPU\|EnsemblGenome=SPU_002503\|UniProtKB=W4XGI8 | W4XGI8 | Uncharacterized protein; unassigned ortholog | SUBFAMILY NOT NAMED (PTHR23430:SF203) | histone(PC00118) | DNA binding | biosynthetic process; cellular process; chromatin organization; nitrogen compound metabolic process; regulation of gene expression, epigenetic; regulation of nucleobase-containing compound metabolic process; transcription, DNA-dependent | intracellular; nuclear chromosome; nucleus |
| STRPU\|EnsemblGenome=SPU_020607\|UniProtKB=W4YXN9 | W4YXN9 | Uncharacterized protein; unassigned ortholog | SEVERIN (PTHR11977:SF85) |  | - | - | - |
| STRPU\|EnsemblGenome=SPU_010221\|UniProtKB=W4Y3D5 | W4Y3D5 | Uncharacterized protein; unassigned ortholog | RAS-LIKE PROTEIN 1 (PTHR24070:SF399) | small GTPase(PC00208) | GTPase activity; protein binding; | cellular process; intracellular protein transport; receptor-mediated endocytosis | - |
| STRPU\|EnsemblGenome=SPU_016849\|UniProtKB=W4YM26 | W4YM26 | Sorting nexin; unassigned ortholog | SORTING NEXIN 6, ISOFORM B (PTHR45850:SF1) |  | - | - | - |
| STRPU\|EnsemblGenome=SPU_017605\|UniProtKB=W4YP78 | W4YP78 | ATP synthase subunit alpha; unassigned ortholog | ATP SYNTHASE SUBUNIT ALPHA, MITOCHONDRIAL (PTHR43089:SF5) | ATP synthase(PC00002);DNA binding protein(PC00009);anion channel(PC00049);hydrolase(PC00121);ligand-gated ion channel(PC00141) | anion channel activity; cation transmembrane transporter activity; hydrolase activity; ligand-gated ion channel activity; proton-transporting ATP synthase activity, rotational mechanism; receptor activity; single-stranded DNA binding | purine nucleobase metabolic process; respiratory electron transport chain | intracellular; proton-transporting ATP synthase complex |
| STRPU\|EnsemblGenome=SPU_013414\|UniProtKB=W4YCD5 | W4YCD5 | Uncharacterized protein; unassigned ortholog | GUANINE NUCLEOTIDE-BINDING PROTEIN G(I) SUBUNIT ALPHA-1 (PTHR10218:SF227) | heterotrimeric G-protein(PC00117) | GTPase activity; adenylate cyclase activity; pyrophosphatase activity; receptor binding; ; signal transducer activity | G-protein coupled receptor signaling pathway; biosynthetic process; catabolic process; cyclic nucleotide metabolic process; intracellular signal transduction; nitrogen compound metabolic process; regulation of catalytic activity; regulation of nucleobase-containing compound metabolic process; regulation of phosphate metabolic process; response to stimulus | heterotrimeric G-protein complex; intracellular; plasma membrane |
| STRPU\|EnsemblGenome=SPU_011345\|UniProtKB=W4Y6J5 | W4Y6J5 | Uncharacterized protein; unassigned ortholog | GH08630P (PTHR14499:SF82) | enzyme modulator(PC00095) | protein binding | cation transport | - |
| STRPU\|EnsemblGenome=SPU_019370\|UniProtKB=W4YU75 | W4YU75 | Uncharacterized protein; unassigned ortholog | SUBFAMILY NOT NAMED (PTHR19331:SF429) | oxidase(PC00175);receptor(PC00197);serine protease(PC00203) | - | - | plasma membrane |
| STRPU\|EnsemblGenome=SPU_014864\|UniProtKB=W4YGH9 | W4YGH9 | Uncharacterized protein; unassigned ortholog | SUBFAMILY NOT NAMED (PTHR19375:SF353) |  | nucleotide binding; protein binding; pyrophosphatase activity | catabolic process; cellular process; nitrogen compound metabolic process; nucleobase-containing compound metabolic process; phosphate-containing compound metabolic process; protein folding; response to abiotic stimulus; response to stress; vesicle-mediated transport | cytosol; nucleus; plasma membrane |
| STRPU\|EnsemblGenome=SPU_020762\|UniProtKB=W4YY40 | W4YY40 | Uncharacterized protein; unassigned ortholog | SUBFAMILY NOT NAMED (PTHR47470:SF1) |  | - | - | - |
| STRPU\|EnsemblGenome=SPU_013834\|UniProtKB=W4YDK4 | W4YDK4 | Uncharacterized protein; unassigned ortholog | 40S RIBOSOMAL PROTEIN S28 (PTHR10769:SF3) | ribosomal protein(PC00202) | RNA binding; structural constituent of ribosome | cellular component biogenesis; cellular process; nitrogen compound metabolic process; organelle organization; rRNA metabolic process | cytosol; organelle; ribosome |
| STRPU\|EnsemblGenome=SPU_013016\|UniProtKB=W4YB92 | W4YB92 | Uncharacterized protein; unassigned ortholog | BCDNA.GH02439 (PTHR11034:SF40) | serine protease(PC00203) | - | - | - |
| STRPU\|EnsemblGenome=SPU_016222\|UniProtKB=W4YKB0 | W4YKB0 | Uncharacterized protein; unassigned ortholog | SUBFAMILY NOT NAMED (PTHR11339:SF373) | protease inhibitor(PC00191) | - | - | - |
| STRPU\|EnsemblGenome=SPU_003185\|UniProtKB=W4XIG0 | W4XIG0 | Uncharacterized protein; unassigned ortholog | CUB AND ZONA PELLUCIDA-LIKE DOMAIN-CONTAINING PROTEIN 1 (PTHR14002:SF20) |  | - | - | - |
| STRPU\|EnsemblGenome=SPU_014660\|UniProtKB=W4YFX5 | W4YFX5 | Uncharacterized protein; unassigned ortholog | ADP/ATP TRANSLOCASE 1 (PTHR45635:SF14) |  | - | - | - |
| STRPU\|EnsemblGenome=SPU_024379\|UniProtKB=W4Z8A7 | W4Z8A7 | 6-phosphogluconate dehydrogenase, decarboxylating; unassigned ortholog | 6-PHOSPHOGLUCONATE DEHYDROGENASE, DECARBOXYLATING (PTHR11811:SF45) | dehydrogenase(PC00092) | oxidoreductase activity | pentose-phosphate shunt | - |
| STRPU\|EnsemblGenome=SPU_020812\|UniProtKB=W4YY90 | W4YY90 | Tubulin alpha chain; unassigned ortholog | TUBULIN ALPHA CHAIN (PTHR11588:SF265) | tubulin(PC00228) | nucleotide binding; structural constituent of cytoskeleton; | cell cycle; cytoskeleton organization | cytoplasm; microtubule |
| STRPU\|EnsemblGenome=SPU_026115\|UniProtKB=W4ZD78 | W4ZD78 | Uncharacterized protein; unassigned ortholog | INTELECTIN-1A-RELATED (PTHR16146:SF42) |  | - | - | - |
| STRPU\|EnsemblGenome=SPU_019691\|UniProtKB=W4YV42 | W4YV42 | Uncharacterized protein; unassigned ortholog | FLOTILLIN-1 (PTHR13806:SF16) |  | - | - | - |
| STRPU\|EnsemblGenome=SPU_000932\|UniProtKB=W4XC29 | W4XC29 | Uncharacterized protein; unassigned ortholog | RAS-RELATED PROTEIN RAB-11B (PTHR24073:SF335) |  | GTPase activity; pyrophosphatase activity | catabolic process; cellular component biogenesis; exocytosis; intracellular protein transport; intracellular signal transduction; nitrogen compound metabolic process; nucleobase-containing compound metabolic process; organelle organization; phagocytosis; phosphate-containing compound metabolic process; protein localization; regulation of biological process; response to endogenous stimulus; | Golgi apparatus; cytoplasm; cytoplasmic membrane-bounded vesicle; endoplasmic reticulum; endosome; mitochondrion; nuclear outer membrane-endoplasmic reticulum membrane network; plasma membrane; vacuole |
| STRPU\|EnsemblGenome=SPU_027060\|UniProtKB=W4ZFV6 | W4ZFV6 | Uncharacterized protein; unassigned ortholog | DRAB5 (PTHR24073:SF858) |  | GTPase activity; pyrophosphatase activity | catabolic process; cellular component biogenesis; exocytosis; intracellular protein transport; intracellular signal transduction; nitrogen compound metabolic process; nucleobase-containing compound metabolic process; organelle organization; phagocytosis; phosphate-containing compound metabolic process; protein localization; regulation of biological process; response to endogenous stimulus; | Golgi apparatus; cytoplasm; cytoplasmic membrane-bounded vesicle; endoplasmic reticulum; endosome; mitochondrion; nuclear outer membrane-endoplasmic reticulum membrane network; plasma membrane; vacuole |
| STRPU\|EnsemblGenome=SPU_014478\|UniProtKB=W4YFE4 | W4YFE4 | Uncharacterized protein; unassigned ortholog | AT07685P-RELATED (PTHR10707:SF10) |  | cation transmembrane transporter activity; hydrogen ion transmembrane transporter activity; oxidoreductase activity | biosynthetic process; cellular process; nitrogen compound metabolic process; nucleobase-containing compound metabolic process; oxidative phosphorylation; phosphate-containing compound metabolic process; respiratory electron transport chain | cytoplasm; mitochondrial inner membrane; protein complex |
| STRPU\|EnsemblGenome=SPU_000241\|UniProtKB=W4XA47 | W4XA47 | Uncharacterized protein; unassigned ortholog | RHEA, ISOFORM B (PTHR19981:SF1) |  | - | - | - |
| STRPU\|EnsemblGenome=SPU_018318\|UniProtKB=W4YR85 | W4YR85 | 60S ribosomal protein L13; unassigned ortholog | 60S RIBOSOMAL PROTEIN L13 (PTHR11722:SF0) | ribosomal protein(PC00202) | RNA binding; structural constituent of ribosome; | - | cytosol; organelle; ribosome |
| STRPU\|EnsemblGenome=SPU_009165\|UniProtKB=W4Y0E3 | W4Y0E3 | Uncharacterized protein; unassigned ortholog | HEAT SHOCK 70 KDA PROTEIN COGNATE 1-RELATED (PTHR19375:SF338) |  | nucleotide binding | catabolic process; cellular process; nitrogen compound metabolic process; nucleobase-containing compound metabolic process; phosphate-containing compound metabolic process; protein folding; response to abiotic stimulus; response to stress; vesicle-mediated transport | cytosol; nucleus; plasma membrane |
| STRPU\|EnsemblGenome=SPU_010829\|UniProtKB=W4Y536 | W4Y536 | Uncharacterized protein; unassigned ortholog | ELONGATION FACTOR 2 (PTHR42908:SF10) | G-protein(PC00020);hydrolase(PC00121);translation elongation factor(PC00222);translation initiation factor(PC00224) | translation elongation factor activity | biosynthetic process; cellular process | - |
| STRPU\|EnsemblGenome=SPU_019458\|UniProtKB=W4YUG2 | W4YUG2 | Uncharacterized protein; unassigned ortholog | RAS-RELATED PROTEIN RAB-2A (PTHR24073:SF354) |  | GTPase activity; pyrophosphatase activity | catabolic process; cellular component biogenesis; exocytosis; intracellular protein transport; intracellular signal transduction; nitrogen compound metabolic process; nucleobase-containing compound metabolic process; organelle organization; phagocytosis; phosphate-containing compound metabolic process; protein localization; regulation of biological process; response to endogenous stimulus; | Golgi apparatus; cytoplasm; cytoplasmic membrane-bounded vesicle; endoplasmic reticulum; mitochondrion; nuclear outer membrane-endoplasmic reticulum membrane network; plasma membrane; vacuole; |
| STRPU\|EnsemblGenome=SPU_003652\|UniProtKB=W4XJS5 | W4XJS5 | Uncharacterized protein; unassigned ortholog | GH09096P (PTHR12422:SF1) |  | - | - | - |
| STRPU\|EnsemblGenome=SPU_020322\|UniProtKB=W4YWW2 | W4YWW2 | Uncharacterized protein; unassigned ortholog | HEAT SHOCK PROTEIN HSP 90-ALPHA-RELATED (PTHR11528:SF34) | Hsp90 family chaperone(PC00028) | - | protein folding; response to stress | - |
| STRPU\|EnsemblGenome=SPU_014711\|UniProtKB=H3IYA6 | H3IYA6 | Histone H3; unassigned ortholog | HISTONE H3.3-RELATED (PTHR11426:SF179) | histone(PC00118) | DNA binding; chromatin binding | DNA metabolic process; cellular component biogenesis; cellular process; chromatin assembly; chromatin organization; nitrogen compound metabolic process | chromosome; intracellular; nucleus; protein-DNA complex |
| STRPU\|EnsemblGenome=SPU_012599\|UniProtKB=W4YA34 | W4YA34 | Uncharacterized protein; unassigned ortholog | INSULIN-LIKE GROWTH FACTOR 2 MRNA-BINDING PROTEIN 2 (PTHR10288:SF93) | enzyme modulator(PC00095);  mRNA splicing factor(PC00148);ribonucleoprotein(PC00201);serine protease(PC00203) | catalytic activity; mRNA binding; protein binding | RNA splicing, via transesterification reactions; induction of apoptosis; intracellular protein transport; mRNA splicing, via spliceosome; nuclear transport; protein metabolic process; signal transduction; transcription from RNA polymerase II promoter | ribonucleoprotein complex |
| STRPU\|EnsemblGenome=SPU_006672\|UniProtKB=W4XTC2 | W4XTC2 | Uncharacterized protein; unassigned ortholog | CALNEXIN 14D-RELATED (PTHR11073:SF1) | calcium-binding protein(PC00060);chaperone(PC00072) | calcium ion binding | exocytosis; intracellular protein transport; protein folding | - |
| STRPU\|EnsemblGenome=SPU_015819\|UniProtKB=W4YJ59 | W4YJ59 | Uncharacterized protein; unassigned ortholog | HOMER, ISOFORM E (PTHR10918:SF1) | signaling molecule(PC00207) | - | - | - |
| STRPU\|EnsemblGenome=SPU_007845\|UniProtKB=W4XWN4 | W4XWN4 | Uncharacterized protein; unassigned ortholog | PROTEIN ENABLED (PTHR11202:SF22) |  | - | - | - |
| STRPU\|EnsemblGenome=SPU_000128\|UniProtKB=W4X9T4 | W4X9T4 | Uncharacterized protein; unassigned ortholog | TROPOMYOSIN-1, ISOFORMS 33/34 (PTHR19269:SF45) |  | actin binding | cellular process; cytoskeleton organization | actin cytoskeleton; intracellular; protein complex |
| STRPU\|EnsemblGenome=SPU_003914\|UniProtKB=W4XKI6 | W4XKI6 | Phospholipid scramblase; unassigned ortholog | PHOSPHOLIPID SCRAMBLASE (PTHR23248:SF56) | transfer/carrier protein(PC00219) | lipid transporter activity | anion transport; biological regulation; cellular component organization; cellular process | - |
| STRPU\|EnsemblGenome=SPU_022528\|UniProtKB=W4Z331 | W4Z331 | Uncharacterized protein; unassigned ortholog | SUBFAMILY NOT NAMED (PTHR19331:SF393) | oxidase(PC00175);receptor(PC00197);serine protease(PC00203) | - | - | - |
| STRPU\|EnsemblGenome=SPU_014869\|UniProtKB=W4YGI4 | W4YGI4 | Uncharacterized protein; unassigned ortholog | PEROXIREDOXIN-1 (PTHR10681:SF111) | peroxidase(PC00180) | oxidoreductase activity; peroxidase activity; | - | - |
| STRPU\|EnsemblGenome=SPU_000881\|UniProtKB=Q5EAJ7 | Q5EAJ7 | Major vault protein; MVP ortholog | MAJOR VAULT PROTEIN (PTHR14165:SF3) | ribonucleoprotein(PC00201) | - | regulation of biological process | cytoplasm; nucleus |
| STRPU\|EnsemblGenome=SPU_025815\|UniProtKB=W4ZCC9 | W4ZCC9 | Sodium/potassium-transporting ATPase subunit alpha; unassigned ortholog | SODIUM/POTASSIUM-TRANSPORTING ATPASE SUBUNIT ALPHA (PTHR43294:SF14) | cation transporter(PC00068);hydrolase(PC00121);ion channel(PC00133) | ATPase activity, coupled to transmembrane movement of substances; cation transmembrane transporter activity; pyrophosphatase activity | catabolic process; cellular process; homeostatic process; nitrogen compound metabolic process; nucleobase-containing compound metabolic process; phosphate-containing compound metabolic process | - |
| STRPU\|EnsemblGenome=SPU_017634\|UniProtKB=W4YPA7 | W4YPA7 | Uncharacterized protein; unassigned ortholog | FILAMIN-A (PTHR38537:SF4) |  | - | - | - |
| STRPU\|EnsemblGenome=SPU_024103\|UniProtKB=W4Z7I4 | W4Z7I4 | Uncharacterized protein; unassigned ortholog | 60 KDA HEAT SHOCK PROTEIN, MITOCHONDRIAL (PTHR45633:SF3) |  | protein binding | cellular process; mitochondrion organization; protein folding | cytosol; mitochondrion; protein complex |
| STRPU\|EnsemblGenome=SPU_012331\|UniProtKB=W4ZBK1 | W4ZBK1 | Histone H4; unassigned ortholog | HISTONE H4 (PTHR10484:SF44) | histone(PC00118) | - | - | intracellular; nuclear chromosome; nucleus; protein-DNA complex |
| STRPU\|EnsemblGenome=SPU_010631\|UniProtKB=W4Y4J1 | W4Y4J1 | Uncharacterized protein; unassigned ortholog | ACTIN-RELATED PROTEIN 3 (PTHR11937:SF31) | actin and actin related protein(PC00039) | structural constituent of cytoskeleton | cellular component organization; cytokinesis; endocytosis; exocytosis; intracellular protein transport; mitosis | actin cytoskeleton; intracellular |
| STRPU\|EnsemblGenome=SPU_027288\|UniProtKB=W4ZGI1 | W4ZGI1 | Uncharacterized protein; unassigned ortholog | SUBFAMILY NOT NAMED (PTHR19331:SF429) | oxidase(PC00175);receptor(PC00197);serine protease(PC00203) | - | - | plasma membrane |
| STRPU\|EnsemblGenome=SPU_022537\|UniProtKB=W4Z340 | W4Z340 | Uncharacterized protein; unassigned ortholog | TRYOSINE 3-MONOOXYGENASE/TRYPTOPHAN 5-MONOOXYGENASE ACTIVATION PROTEIN, ZETA POLYPEPTIDE-RELATED (PTHR18860:SF66) | chaperone(PC00072) | - | cell cycle; signal transduction | - |
| STRPU\|EnsemblGenome=SPU_027378\|UniProtKB=W4ZGS0 | W4ZGS0 | Phospholipid scramblase; unassigned ortholog | PHOSPHOLIPID SCRAMBLASE (PTHR23248:SF56) | transfer/carrier protein(PC00219) | lipid transporter activity | anion transport; biological regulation; cellular component organization; cellular process | - |
| STRPU\|EnsemblGenome=SPU_011065\|UniProtKB=W4Y5R7 | W4Y5R7 | Integrin beta; unassigned ortholog | INTEGRIN BETA-PS (PTHR10082:SF41) | cell adhesion molecule(PC00069);receptor(PC00197) | receptor binding | cell surface receptor signaling pathway; cell-matrix adhesion; cellular component movement; localization; locomotion; regulation of biological process; response to stimulus | cell junction; integral to membrane; plasma membrane; protein complex |
| STRPU\|EnsemblGenome=SPU_027236\|UniProtKB=W4ZGC9 | W4ZGC9 | Uncharacterized protein; unassigned ortholog | GH26960P-RELATED (PTHR11743:SF23) | anion channel(PC00049);voltage-gated ion channel(PC00241) | anion channel activity; voltage-gated ion channel activity; | anion transport | - |
| STRPU\|EnsemblGenome=SPU_013413\|UniProtKB=H3IH34 | H3IH34 | Uncharacterized protein; unassigned ortholog | PTERIN-4-ALPHA-CARBINOLAMINE DEHYDRATASE 2 (PTHR12599:SF0) | dehydratase(PC00091) | hydro-lyase activity | transcription from RNA polymerase II promoter | - |
| STRPU\|EnsemblGenome=SPU_025555\|UniProtKB=W4ZBM5 | W4ZBM5 | Uncharacterized protein; unassigned ortholog | ADP-RIBOSYLATION FACTOR 6A (PTHR11711:SF322) |  | - | cellular process; intracellular protein transport; protein localization; vesicle-mediated transport | - |
| STRPU\|EnsemblGenome=SPU_011157\|UniProtKB=W4Y609 | W4Y609 | Uncharacterized protein; unassigned ortholog | RAS-RELATED PROTEIN RAB-14 (PTHR24073:SF185) |  | GTPase activity; pyrophosphatase activity | catabolic process; cellular component biogenesis; exocytosis; intracellular protein transport; intracellular signal transduction; nitrogen compound metabolic process; nucleobase-containing compound metabolic process; organelle organization; phagocytosis; phosphate-containing compound metabolic process; protein localization; regulation of biological process; response to endogenous stimulus; | Golgi apparatus; cytoplasm; cytoplasmic membrane-bounded vesicle; endoplasmic reticulum; mitochondrion; nuclear outer membrane-endoplasmic reticulum membrane network; plasma membrane; vacuole; |
| STRPU\|EnsemblGenome=SPU_021394\|UniProtKB=W4YZW1 | W4YZW1 | Uncharacterized protein; unassigned ortholog | AT21416P (PTHR11216:SF31) | G-protein modulator(PC00022);calcium-binding protein(PC00060);membrane traffic protein(PC00150) | calcium ion binding; protein binding; small GTPase regulator activity | cellular process; endocytosis; intracellular protein transport | - |
| STRPU\|EnsemblGenome=SPU_016790\|UniProtKB=A0A0B4J2V7 | A0A0B4J2V7 | Uncharacterized protein; unassigned ortholog | RAS-RELATED PROTEIN RAC1-RELATED (PTHR24072:SF275) | small GTPase(PC00208) | GTPase activity | catabolic process; intracellular signal transduction; nitrogen compound metabolic process; nucleobase-containing compound metabolic process; phosphate-containing compound metabolic process; regulation of biological process; response to stimulus | - |
| STRPU\|EnsemblGenome=SPU_008560\|UniProtKB=W4XYP1 | W4XYP1 | Uncharacterized protein; unassigned ortholog | 78 KDA GLUCOSE-REGULATED PROTEIN (PTHR19375:SF144) |  | nucleotide binding; protein binding; pyrophosphatase activity | catabolic process; nitrogen compound metabolic process; nucleobase-containing compound metabolic process; phosphate-containing compound metabolic process; protein folding; proteolysis; regulation of biological process; response to abiotic stimulus; response to stress; signal transduction | cytoplasm; endoplasmic reticulum; nucleus; protein complex |
| STRPU\|EnsemblGenome=SPU_007036\|UniProtKB=W4XUD3 | W4XUD3 | Uncharacterized protein; unassigned ortholog | VACUOLAR PROTEIN SORTING-ASSOCIATED PROTEIN 35 (PTHR11099:SF0) | membrane traffic protein(PC00150) | transporter activity | cellular process; intracellular protein transport; protein localization; vesicle-mediated transport | cytoplasm; endosome; membrane; protein complex |
| STRPU\|EnsemblGenome=SPU_026843\|UniProtKB=W4ZF96 | W4ZF96 | Uncharacterized protein; unassigned ortholog | SUBFAMILY NOT NAMED (PTHR24543:SF303) | cell adhesion molecule(PC00069);hydrolase(PC00121);membrane-bound signaling molecule(PC00152);receptor(PC00197) | - | - | - |
| STRPU\|EnsemblGenome=SPU_021303\|UniProtKB=Q4JQQ3 | Q4JQQ3 | Calcium-transporting ATPase; PMCA ortholog | PLASMA MEMBRANE CALCIUM-TRANSPORTING ATPASE 4 (PTHR24093:SF369) | cation transporter(PC00068);hydrolase(PC00121);ion channel(PC00133) | ATPase activity, coupled to transmembrane movement of substances; cation transmembrane transporter activity; pyrophosphatase activity | catabolic process; cellular process; nitrogen compound metabolic process; nucleobase-containing compound metabolic process; phosphate-containing compound metabolic process | integral to membrane; intracellular; organelle; plasma membrane |
| STRPU\|EnsemblGenome=SPU_007553\|UniProtKB=W4XVU5 | W4XVU5 | Uncharacterized protein; unassigned ortholog | GUANINE NUCLEOTIDE-BINDING PROTEIN G(O) SUBUNIT ALPHA (PTHR10218:SF293) | heterotrimeric G-protein(PC00117) | GTPase activity; adenylate cyclase activity; pyrophosphatase activity; receptor binding; signal transducer activity | G-protein coupled receptor signaling pathway; biosynthetic process; catabolic process; cyclic nucleotide metabolic process; intracellular signal transduction; nitrogen compound metabolic process; regulation of catalytic activity; regulation of nucleobase-containing compound metabolic process; regulation of phosphate metabolic process; response to stimulus | heterotrimeric G-protein complex; intracellular; plasma membrane |
| STRPU\|EnsemblGenome=SPU_014994\|UniProtKB=W4YGV4 | W4YGV4 | Uncharacterized protein; unassigned ortholog | SUBFAMILY NOT NAMED (PTHR19331:SF261) | oxidase(PC00175);receptor(PC00197);serine protease(PC00203) | - | - | - |
| STRPU\|EnsemblGenome=SPU_018624\|UniProtKB=W4YS38 | W4YS38 | Malate dehydrogenase; unassigned ortholog | MALATE DEHYDROGENASE, MITOCHONDRIAL (PTHR11540:SF16) | dehydrogenase(PC00092) | oxidoreductase activity | carbohydrate metabolic process; generation of precursor metabolites and energy; tricarboxylic acid cycle | - |
| STRPU\|EnsemblGenome=SPU_026996\|UniProtKB=W4ZFP7 | W4ZFP7 | Uncharacterized protein; unassigned ortholog | GOLGI APPARATUS PROTEIN 1 (PTHR11884:SF1) |  | - | - | - |
| STRPU\|EnsemblGenome=SPU_013100\|UniProtKB=W4YBH6 | W4YBH6 | Uncharacterized protein; unassigned ortholog | SUBFAMILY NOT NAMED (PTHR42693:SF15) | hydrolase(PC00121) | hydrolase activity | phospholipid metabolic process; polysaccharide metabolic process; sulfur compound metabolic process | - |
| STRPU\|EnsemblGenome=SPU_026637\|UniProtKB=W4ZEP3 | W4ZEP3 | Uncharacterized protein; unassigned ortholog | SUBFAMILY NOT NAMED (PTHR46893:SF1) |  | - | - | - |
| STRPU\|EnsemblGenome=SPU_010762\|UniProtKB=W4Y4X0 | W4Y4X0 | Uncharacterized protein; unassigned ortholog | RAS-RELATED PROTEIN RAB-1A (PTHR24073:SF212) |  | GTPase activity; pyrophosphatase activity | catabolic process; cellular component biogenesis; exocytosis; intracellular protein transport; intracellular signal transduction; nitrogen compound metabolic process; nucleobase-containing compound metabolic process; organelle organization; phagocytosis; phosphate-containing compound metabolic process; protein localization; regulation of biological process; response to endogenous stimulus; | Golgi apparatus; cytoplasm; cytoplasmic membrane-bounded vesicle; endoplasmic reticulum; mitochondrion; nuclear outer membrane-endoplasmic reticulum membrane network; plasma membrane; vacuole; |
| STRPU\|EnsemblGenome=SPU_013010\|UniProtKB=W4YB86 | W4YB86 | Glutamate dehydrogenase; unassigned ortholog | GLUTAMATE DEHYDROGENASE 1, MITOCHONDRIAL (PTHR11606:SF13) | dehydrogenase(PC00092) | oxidoreductase activity | cellular amino acid catabolic process | - |
| STRPU\|EnsemblGenome=SPU_023217\|UniProtKB=W4Z513 | W4Z513 | GTP-binding nuclear protein; unassigned ortholog | GTP-BINDING NUCLEAR PROTEIN RAN (PTHR24071:SF0) | small GTPase(PC00208) | GTPase activity; pyrophosphatase activity | RNA localization; catabolic process; cellular component biogenesis; cellular process; nitrogen compound metabolic process; nuclear transport; nucleobase-containing compound metabolic process; nucleobase-containing compound transport; phosphate-containing compound metabolic process; protein localization; protein targeting | cytoplasm; nucleus |
| STRPU\|EnsemblGenome=SPU_021007\|UniProtKB=W4YYT1 | W4YYT1 | Uncharacterized protein; unassigned ortholog | F-ACTIN-CAPPING PROTEIN SUBUNIT BETA (PTHR10619:SF0) | non-motor actin binding protein(PC00165) | actin binding | cellular component morphogenesis; cellular process; cytoskeleton organization; protein complex assembly; regulation of biological process | actin cytoskeleton; intracellular; protein complex |
| STRPU\|EnsemblGenome=SPU_011029\|UniProtKB=A0A0B4J2V0 | A0A0B4J2V0 | Uncharacterized protein; unassigned ortholog | RAS-LIKE GTP-BINDING PROTEIN RHO1 (PTHR24072:SF168) | small GTPase(PC00208) | GTPase activity; nucleotide binding; protein binding; pyrophosphatase activity; signal transducer activity | catabolic process; cell cycle; cellular component biogenesis; cellular component morphogenesis; cellular component movement; cytokinesis; cytoskeleton organization; intracellular signal transduction; localization; locomotion; nitrogen compound metabolic process; nucleobase-containing compound metabolic process; phosphate-containing compound metabolic process; regulation of biological process | cytoplasm; organelle; plasma membrane |
| STRPU\|EnsemblGenome=SPU_018505\|UniProtKB=W4YRS2 | W4YRS2 | Uncharacterized protein; unassigned ortholog | GUANINE NUCLEOTIDE-BINDING PROTEIN SUBUNIT BETA-1 (PTHR19850:SF25) | heterotrimeric G-protein(PC00117);hydrolase(PC00121) | GTPase activity; protein binding; | cellular process | heterotrimeric G-protein complex |
| STRPU\|EnsemblGenome=SPU_020875\|UniProtKB=W4YYF1 | W4YYF1 | Uncharacterized protein; unassigned ortholog | SUBFAMILY NOT NAMED (PTHR12546:SF56) | membrane traffic protein(PC00150) | - | - | - |
| STRPU\|EnsemblGenome=SPU_012282\|UniProtKB=W4Y969 | W4Y969 | Uncharacterized protein; unassigned ortholog | ACTIN, CYTOPLASMIC 1-RELATED (PTHR11937:SF155) | actin and actin related protein(PC00039) | structural constituent of cytoskeleton | cellular component organization; cytokinesis; endocytosis; exocytosis; intracellular protein transport; mitosis | actin cytoskeleton; intracellular |
| STRPU\|EnsemblGenome=SPU_024221\|UniProtKB=W4Z7V2 | W4Z7V2 | Uncharacterized protein; unassigned ortholog | FI18122P1 (PTHR10555:SF170) | membrane trafficking regulatory protein(PC00151) | lipid binding | cellular process; endocytosis; organelle organization | cytoplasm; endosome; membrane |
| STRPU\|EnsemblGenome=SPU_022357\|UniProtKB=W4Z2L4 | W4Z2L4 | Uncharacterized protein; unassigned ortholog | EXPORTIN 7 (PTHR12596:SF2) | transfer/carrier protein(PC00219) | transporter activity | cellular process; intracellular protein transport; nuclear transport; protein localization | cytoplasm; integral to membrane; nuclear envelope; protein complex |
| STRPU\|EnsemblGenome=SPU_013480\|UniProtKB=W4YCK1 | W4YCK1 | Uncharacterized protein; unassigned ortholog | GH25853P (PTHR19871:SF14) |  | - | - | - |
| STRPU\|EnsemblGenome=SPU_009190\|UniProtKB=W4Y0G8 | W4Y0G8 | Uncharacterized protein; unassigned ortholog | ALPHA-ENDOSULFINE (PTHR10358:SF6) |  | phosphatase inhibitor activity; phosphoprotein phosphatase activity; | cellular process; cellular protein modification process; regulation of phosphate metabolic process | cytoplasm |
| STRPU\|EnsemblGenome=SPU_019378\|UniProtKB=W4YU83 | W4YU83 | Phosphotransferase; unassigned ortholog | HEXOKINASE 1 (PTHR19443:SF16) |  | carbohydrate kinase activity | catabolic process; cellular glucose homeostasis; cellular process; glycolysis; phosphate-containing compound metabolic process | cytosol |
| STRPU\|EnsemblGenome=SPU_020149\|UniProtKB=W4YWE1 | W4YWE1 | Dolichyl-diphosphooligosaccharide--protein glycosyltransferase subunit 1; unassigned ortholog | DOLICHYL-DIPHOSPHOOLIGOSACCHARIDE--PROTEIN GLYCOSYLTRANSFERASE SUBUNIT 1 (PTHR21049:SF0) | glycosyltransferase(PC00111) | transferase activity, transferring glycosyl groups | biosynthetic process; carbohydrate metabolic process; cellular process; protein glycosylation | cytoplasm; endoplasmic reticulum; nuclear outer membrane-endoplasmic reticulum membrane network; protein complex |
| STRPU\|EnsemblGenome=SPU_009066\|UniProtKB=W4Y044 | W4Y044 | Purine nucleoside phosphorylase; unassigned ortholog | S-METHYL-5'-THIOADENOSINE PHOSPHORYLASE (PTHR42679:SF2) | phosphorylase(PC00187) | transferase activity, transferring glycosyl groups | biosynthetic process; cellular amino acid biosynthetic process; cellular process; nitrogen compound metabolic process; sulfur compound metabolic process | cytosol |
| STRPU\|EnsemblGenome=SPU_000508\|UniProtKB=W4XAW2 | W4XAW2 | Uncharacterized protein; unassigned ortholog | GUANINE NUCLEOTIDE-BINDING PROTEIN SUBUNIT BETA-1 (PTHR19850:SF25) | heterotrimeric G-protein(PC00117);hydrolase(PC00121) | GTPase activity; protein binding; | cellular process | heterotrimeric G-protein complex |
| STRPU\|EnsemblGenome=SPU_011961\|UniProtKB=W4Y8A2 | W4Y8A2 | Uncharacterized protein; unassigned ortholog | RAS-RELATED PROTEIN RAB-21 (PTHR24073:SF509) |  | GTPase activity | catabolic process; cellular component biogenesis; exocytosis; intracellular protein transport; intracellular signal transduction; nitrogen compound metabolic process; nucleobase-containing compound metabolic process; organelle organization; phagocytosis; phosphate-containing compound metabolic process; protein localization; regulation of biological process; response to endogenous stimulus; | Golgi apparatus; cytoplasm; cytoplasmic membrane-bounded vesicle; endoplasmic reticulum; endosome; mitochondrion; nuclear outer membrane-endoplasmic reticulum membrane network; plasma membrane; vacuole |
| STRPU\|EnsemblGenome=SPU_014568\|UniProtKB=W4YFN2 | W4YFN2 | Uncharacterized protein; unassigned ortholog | AT06885P-RELATED (PTHR10264:SF118) | cytoskeletal protein(PC00085);protease inhibitor(PC00191) | structural constituent of cytoskeleton | - | cytoskeleton; intracellular |
| STRPU\|EnsemblGenome=SPU_001817\|UniProtKB=W4XEK3 | W4XEK3 | Pyruvate kinase; unassigned ortholog | PYRUVATE KINASE PKM (PTHR11817:SF3) |  | kinase activity | catabolic process; cellular process; glycolysis; phosphate-containing compound metabolic process | cytoplasm |
| STRPU\|EnsemblGenome=SPU_004628\|UniProtKB=W4XMJ7 | W4XMJ7 | Uncharacterized protein; unassigned ortholog | MICROTUBULE-ASSOCIATED PROTEIN FUTSCH (PTHR13843:SF12) | non-motor microtubule binding protein(PC00166) | actin binding; microtubule binding | cell differentiation; cellular component morphogenesis; cellular process; cytoskeleton organization; nervous system development; regulation of biological process; single-multicellular organism process | cytosol; dendrite; microtubule; neuronal cell body |
| STRPU\|EnsemblGenome=SPU_017814\|UniProtKB=W4YPT6 | W4YPT6 | Uncharacterized protein; unassigned ortholog | CALMODULIN, STRIATED MUSCLE (PTHR23050:SF245) | calmodulin(PC00061) | calcium ion binding; calmodulin binding; | cell cycle; cellular component movement | - |
| STRPU\|EnsemblGenome=SPU_005572\|UniProtKB=W4XQ85 | W4XQ85 | Uncharacterized protein; unassigned ortholog | HIGH MOBILITY GROUP PROTEIN DSP1 (PTHR13711:SF302) | HMG box transcription factor(PC00024);chromatin/chromatin-binding protein(PC00077);signaling molecule(PC00207) | chromatin binding | biosynthetic process; cellular process; chromatin organization; chromatin remodeling; nitrogen compound metabolic process; regulation of nucleobase-containing compound metabolic process; transcription, DNA-dependent | intracellular; nucleus |
| STRPU\|EnsemblGenome=SPU_011106\|UniProtKB=W4Y5V8 | W4Y5V8 | Annexin; unassigned ortholog | ANNEXIN A13 (PTHR10502:SF175) |  | - | fatty acid metabolic process | - |
| STRPU\|EnsemblGenome=SPU_000236\|UniProtKB=W4XA42 | W4XA42 | Isocitrate dehydrogenase [NADP]; unassigned ortholog | ISOCITRATE DEHYDROGENASE [NADP] CYTOPLASMIC (PTHR11822:SF21) |  | - | - | - |
| STRPU\|EnsemblGenome=SPU_005779\|UniProtKB=W4XQT8 | W4XQT8 | Uncharacterized protein; unassigned ortholog | RAS-RELATED PROTEIN RAP-1A (PTHR24070:SF395) | small GTPase(PC00208) | GTPase activity; protein binding | G-protein coupled receptor signaling pathway; I-kappaB kinase/NF-kappaB cascade; MAPK cascade; cell adhesion; intracellular protein transport; neurological system process; receptor-mediated endocytosis; synaptic transmission | - |
| STRPU\|EnsemblGenome=SPU_026183\|UniProtKB=W4ZDE4 | W4ZDE4 | Uncharacterized protein; unassigned ortholog | NA/CA EXCHANGERS (PTHR11878:SF45) |  | - | - | - |
| STRPU\|EnsemblGenome=SPU_009549\|UniProtKB=W4Y1G9 | W4Y1G9 | Uncharacterized protein; unassigned ortholog | SUBFAMILY NOT NAMED (PTHR12345:SF3) | membrane trafficking regulatory protein(PC00151) | - | regulation of biological process | cytoplasm; plasma membrane |
| STRPU\|GeneID=581500\|UniProtKB=W4XTP5 | W4XTP5 | Uncharacterized protein; unassigned ortholog | SUBFAMILY NOT NAMED (PTHR11937:SF381) | actin and actin related protein(PC00039) | structural constituent of cytoskeleton | cellular component organization; cytokinesis; endocytosis; exocytosis; intracellular protein transport; mitosis | actin cytoskeleton; intracellular |
| STRPU\|EnsemblGenome=SPU_016414\|UniProtKB=W4YKU8 | W4YKU8 | Uncharacterized protein; unassigned ortholog | V-TYPE PROTON ATPASE SUBUNIT B (PTHR43389:SF4) | ATP synthase(PC00002);DNA binding protein(PC00009);anion channel(PC00049);hydrolase(PC00121);ligand-gated ion channel(PC00141) | anion channel activity; cation transmembrane transporter activity; hydrolase activity; ligand-gated ion channel activity; proton-transporting ATP synthase activity, rotational mechanism; receptor activity; single-stranded DNA binding | nucleobase-containing compound transport; purine nucleobase metabolic process; respiratory electron transport chain | intracellular; proton-transporting ATP synthase complex |
| STRPU\|EnsemblGenome=SPU_016831\|UniProtKB=W4YM08 | W4YM08 | Serine hydroxymethyltransferase; unassigned ortholog | SERINE HYDROXYMETHYLTRANSFERASE, MITOCHONDRIAL (PTHR11680:SF17) | methyltransferase(PC00155) | methyltransferase activity | cellular amino acid metabolic process; nucleobase-containing compound metabolic process | - |
| STRPU\|EnsemblGenome=SPU_026031\|UniProtKB=W4ZCZ4 | W4ZCZ4 | Uncharacterized protein; unassigned ortholog | SI:DKEY-167I21.2 (PTHR12021:SF16) |  | - | - | - |
| STRPU\|EnsemblGenome=SPU_016758\|UniProtKB=W4YLT7 | W4YLT7 | Nucleoside diphosphate kinase; unassigned ortholog | NUCLEOSIDE DIPHOSPHATE KINASE (PTHR11349:SF91) |  | kinase activity | biosynthetic process; cellular process; nitrogen compound metabolic process; nucleobase-containing compound metabolic process; phosphate-containing compound metabolic process | - |
| STRPU\|EnsemblGenome=SPU_013819\|UniProtKB=W4YDI9 | W4YDI9 | Uncharacterized protein; unassigned ortholog | ENOLASE (PTHR11902:SF1) |  | - | - | - |
| STRPU\|EnsemblGenome=SPU_005061\|UniProtKB=W4XNS9 | W4XNS9 | Uncharacterized protein; unassigned ortholog | FLOTILLIN-2 (PTHR13806:SF20) |  | - | - | - |
| STRPU\|EnsemblGenome=SPU_025962\|UniProtKB=W4ZCS5 | W4ZCS5 | Annexin; unassigned ortholog | ANNEXIN A7 (PTHR10502:SF140) |  | - | fatty acid metabolic process | - |
| STRPU\|EnsemblGenome=SPU_002788\|UniProtKB=W4XHC0 | W4XHC0 | Tubulin beta chain; unassigned ortholog | TUBULIN BETA-1 CHAIN (PTHR11588:SF293) | tubulin(PC00228) | nucleotide binding; structural constituent of cytoskeleton; | cell cycle; cytoskeleton organization | cytoplasm; microtubule |
| STRPU\|EnsemblGenome=SPU_024525\|UniProtKB=W4Z8Q1 | W4Z8Q1 | Tyrosine-protein kinase; unassigned ortholog | TYROSINE-PROTEIN KINASE SRC64B (PTHR24418:SF373) |  | receptor binding | cell surface receptor signaling pathway; immune response; regulation of biological process | - |
| STRPU\|EnsemblGenome=SPU_027527\|UniProtKB=W4ZH65 | W4ZH65 | Uncharacterized protein; unassigned ortholog | ENDOPLASMIN (PTHR11528:SF54) | Hsp90 family chaperone(PC00028) | - | protein folding; response to stress | - |
| STRPU\|EnsemblGenome=SPU_019087\|UniProtKB=W4YTE5 | W4YTE5 | Uncharacterized protein; unassigned ortholog | RAB FAMILY (PTHR24073:SF57) |  | GTPase activity; pyrophosphatase activity | catabolic process; cellular component biogenesis; exocytosis; intracellular protein transport; intracellular signal transduction; nitrogen compound metabolic process; nucleobase-containing compound metabolic process; organelle organization; phagocytosis; phosphate-containing compound metabolic process; protein localization; regulation of biological process; response to endogenous stimulus; | Golgi apparatus; cytoplasm; cytoplasmic membrane-bounded vesicle; endoplasmic reticulum; mitochondrion; nuclear outer membrane-endoplasmic reticulum membrane network; plasma membrane; vacuole; |
| STRPU\|EnsemblGenome=SPU_002012\|UniProtKB=W4XF48 | W4XF48 | Uncharacterized protein; unassigned ortholog | ATPASE INHIBITOR, MITOCHONDRIAL (PTHR23407:SF6) | ligase(PC00142) | enzyme inhibitor activity; ligase activity; protein binding; pyrophosphatase activity | biosynthetic process; catabolic process; cellular amino acid biosynthetic process; cellular process; coenzyme metabolic process; porphyrin-containing compound metabolic process; pteridine-containing compound metabolic process; regulation of nucleobase-containing compound metabolic process; regulation of phosphate metabolic process | cytoplasm; mitochondrion |
| STRPU\|EnsemblGenome=SPU_026078\|UniProtKB=W4ZD41 | W4ZD41 | Uncharacterized protein; unassigned ortholog | ACTIN, CYTOPLASMIC 1-RELATED (PTHR11937:SF155) | actin and actin related protein(PC00039) | structural constituent of cytoskeleton | cellular component organization; cytokinesis; endocytosis; exocytosis; intracellular protein transport; mitosis | actin cytoskeleton; intracellular |
| STRPU\|EnsemblGenome=SPU_001751\|UniProtKB=W4XED8 | W4XED8 | Uncharacterized protein; unassigned ortholog | ZGC:110239 (PTHR12411:SF575) | cysteine protease(PC00081);protease inhibitor(PC00191) | cysteine-type peptidase activity; enzyme activator activity | apoptotic process; catabolic process; proteolysis; regulation of biological process; response to stimulus; signal transduction | cytoplasm; extracellular space; lysosome; vacuole |
| STRPU\|EnsemblGenome=SPU_019494\|UniProtKB=A0A0B4J2W0 | A0A0B4J2W0 | Uncharacterized protein; unassigned ortholog | CDC42 HOMOLOG (PTHR24072:SF192) | small GTPase(PC00208) | GTPase activity; nucleotide binding; protein binding; pyrophosphatase activity; signal transducer activity | catabolic process; cellular component morphogenesis; cytoskeleton organization; intracellular signal transduction; nitrogen compound metabolic process; nucleobase-containing compound metabolic process; phosphate-containing compound metabolic process; regulation of biological process; response to stimulus | cell projection; cytoplasm; cytoskeleton; plasma membrane |
| STRPU\|EnsemblGenome=SPU_016882\|UniProtKB=W4YM59 | W4YM59 | Uncharacterized protein; unassigned ortholog | V-TYPE PROTON ATPASE CATALYTIC SUBUNIT A (PTHR43607:SF1) | ATP synthase(PC00002);DNA binding protein(PC00009);anion channel(PC00049);ligand-gated ion channel(PC00141);protease(PC00190) | ATPase activity, coupled to transmembrane movement of substances; cation transmembrane transporter activity; hydrogen ion transmembrane transporter activity; pyrophosphatase activity; | catabolic process; cellular process; nitrogen compound metabolic process; nucleobase-containing compound metabolic process; phosphate-containing compound metabolic process | cytoplasm; plasma membrane; vacuole |
| STRPU\|EnsemblGenome=SPU_024704\|UniProtKB=W4Z979 | W4Z979 | Uncharacterized protein; unassigned ortholog | SPLICEOSOME RNA HELICASE DDX39B (PTHR24031:SF605) |  | - | - | - |
| STRPU\|EnsemblGenome=SPU_013452\|UniProtKB=W4YCH3 | W4YCH3 | Uncharacterized protein; unassigned ortholog | 40S RIBOSOMAL PROTEIN SA (PTHR11489:SF9) |  | structural constituent of ribosome | RNA localization; biosynthetic process; cellular component biogenesis; cellular process; nitrogen compound metabolic process; nuclear transport; nucleobase-containing compound transport; organelle organization; rRNA metabolic process; translation | cytosol; organelle; ribosome |
| STRPU\|EnsemblGenome=SPU_020920\|UniProtKB=W4YYJ5 | W4YYJ5 | Uncharacterized protein; unassigned ortholog | ALPHA-ACTININ, SARCOMERIC (PTHR11915:SF435) |  | - | - | - |
| STRPU\|EnsemblGenome=SPU_024188\|UniProtKB=W4Z7R9 | W4Z7R9 | Annexin; unassigned ortholog | ANNEXIN A13 (PTHR10502:SF175) |  | - | fatty acid metabolic process | - |
| STRPU\|EnsemblGenome=SPU_002356\|UniProtKB=W4XG43 | W4XG43 | V-type proton ATPase subunit G; unassigned ortholog | V-TYPE PROTON ATPASE SUBUNIT G (PTHR12713:SF11) | ATP synthase(PC00002);hydrolase(PC00121) | cation transmembrane transporter activity; hydrolase activity; proton-transporting ATP synthase activity, rotational mechanism | nucleobase-containing compound metabolic process | intracellular; proton-transporting ATP synthase complex |
| STRPU\|EnsemblGenome=SPU_004785\|UniProtKB=W4XN05 | W4XN05 | Rab GDP dissociation inhibitor; unassigned ortholog | RAB GDP DISSOCIATION INHIBITOR (PTHR11787:SF8) | G-protein modulator(PC00022);acyltransferase(PC00042) | protein binding | cellular process; intracellular protein transport; vesicle-mediated transport | - |
| STRPU\|Gene=H2BL4_STRPU\|UniProtKB=P16890 | P16890 | Late histone H2B.L4 (Fragment); unassigned ortholog | SUBFAMILY NOT NAMED (PTHR23428:SF70) | histone(PC00118) | DNA binding | DNA metabolic process; cellular component biogenesis; cellular process; chromatin assembly; chromatin organization; nitrogen compound metabolic process | - |
| STRPU\|EnsemblGenome=SPU_019667\|UniProtKB=W4YV18 | W4YV18 | Uncharacterized protein; unassigned ortholog | ADENOSINE DEAMINASE CECR1 (PTHR11409:SF39) | deaminase(PC00088);growth factor(PC00112) | deaminase activity; hydrolase activity | biosynthetic process; catabolic process; cellular process; nitrogen compound metabolic process; purine nucleobase metabolic process | cytosol |
| STRPU\|EnsemblGenome=SPU_001613\|UniProtKB=W4XE04 | W4XE04 | Uncharacterized protein; unassigned ortholog | FI08416P-RELATED (PTHR23048:SF0) | actin family cytoskeletal protein(PC00041);calmodulin(PC00061) | calcium ion binding; calmodulin binding; structural constituent of cytoskeleton | - | actin cytoskeleton; intracellular |
| STRPU\|EnsemblGenome=SPU_000513\|UniProtKB=W4XAW7 | W4XAW7 | Uncharacterized protein; unassigned ortholog | DUAL OXIDASE (PTHR11972:SF126) | oxidase(PC00175) | oxidoreductase activity | immune system process; respiratory electron transport chain | - |
| STRPU\|EnsemblGenome=SPU_010946\|UniProtKB=Q6QM19 | Q6QM19 | Guanine nucleotide-binding protein G(12) alpha subunit; unassigned ortholog | GUANINE NUCLEOTIDE-BINDING PROTEIN SUBUNIT ALPHA HOMOLOG (PTHR10218:SF205) | heterotrimeric G-protein(PC00117) | GTPase activity; adenylate cyclase activity; pyrophosphatase activity; receptor binding; signal transducer activity | G-protein coupled receptor signaling pathway; biosynthetic process; catabolic process; cyclic nucleotide metabolic process; intracellular signal transduction; nitrogen compound metabolic process; regulation of catalytic activity; regulation of nucleobase-containing compound metabolic process; regulation of phosphate metabolic process; response to stimulus | heterotrimeric G-protein complex; intracellular; plasma membrane |
| STRPU\|EnsemblGenome=SPU_018932\|UniProtKB=W4YSZ3 | W4YSZ3 | Uncharacterized protein; unassigned ortholog | SUBFAMILY NOT NAMED (PTHR23348:SF16) |  | RNA binding | RNA splicing; cellular process; nitrogen compound metabolic process; regulation of nucleobase-containing compound metabolic process | cytoplasm; plasma membrane |
| STRPU\|EnsemblGenome=SPU_002961\|UniProtKB=W4XHT9 | W4XHT9 | Uncharacterized protein; unassigned ortholog | DYNAMIN (PTHR11566:SF144) | hydrolase(PC00121);microtubule family cytoskeletal protein(PC00157);small GTPase(PC00208) | GTPase activity; microtubule binding; pyrophosphatase activity | catabolic process; cellular process; nitrogen compound metabolic process; nucleobase-containing compound metabolic process; organelle organization; phosphate-containing compound metabolic process | cytoplasm; peroxisome; plastid |
| STRPU\|EnsemblGenome=SPU_023184\|UniProtKB=W4Z4Y0 | W4Z4Y0 | Uncharacterized protein; unassigned ortholog | SUBFAMILY NOT NAMED (PTHR24543:SF287) | cell adhesion molecule(PC00069);  hydrolase(PC00121);  membrane-bound signaling molecule(PC00152);  receptor(PC00197) | - | - | - |
| STRPU\|Gene=FASC_STRPU\|UniProtKB=Q05634 | Q05634 | Fascin; unassigned ortholog | FASCIN (PTHR10551:SF9) | non-motor actin binding protein(PC00165) | actin binding | cellular component biogenesis; cellular component movement; cytoskeleton organization; localization; locomotion | actin cytoskeleton; cytoplasm; protein complex |
| STRPU\|EnsemblGenome=SPU_015276\|UniProtKB=W4YHN1 | W4YHN1 | Uncharacterized protein; unassigned ortholog | UBIQUITIN-60S RIBOSOMAL PROTEIN L40 (PTHR10666:SF272) | ribosomal protein(PC00202) | RNA binding; protein binding; structural constituent of ribosome; | catabolic process; cellular process; cellular protein modification process; proteolysis | cytosol; nucleus; ribosome |
| STRPU\|EnsemblGenome=SPU_012743\|UniProtKB=W4YAH5 | W4YAH5 | Uncharacterized protein; unassigned ortholog | SUBFAMILY NOT NAMED (PTHR42693:SF15) | hydrolase(PC00121) | hydrolase activity; | phospholipid metabolic process; polysaccharide metabolic process; sulfur compound metabolic process | - |
| STRPU\|EnsemblGenome=SPU_011119\|UniProtKB=W4Y5X1 | W4Y5X1 | Glucose-6-phosphate isomerase; unassigned ortholog | GLUCOSE-6-PHOSPHATE ISOMERASE (PTHR11469:SF4) | isomerase(PC00135) | isomerase activity | gluconeogenesis; glycolysis | - |
