# Supplementary material for: Changes in the proteome of sea urchin Paracentrotus lividus coelomocytes in response to LPS injection into the body cavity
Source: PLoS One. 2020 Feb 19;15(2):e0228893. doi: 10.1371/journal.pone.0228893 (PMC7030939; doi:10.1371/journal.pone.0228893)
Supplement: S2 Table — Conversion table between UNIPROT and STRING identifiers. Where possible, protein name, gene name, gene symbol and ortholog have been added. (DOCX) [file pone.0228893.s002.docx]

**S2 Table**

| **UNIPROT ID** | **PROTEIN NAME; GENE NAME; GENE SYMBOL; ORTHOLOG** | **STRING ID** |
| --- | --- | --- |
| W4Y4J1 | Actin related protein 3; ARP3; unassigned ortholog | LOC576708 |
| W4XAW7 | Uncharacterized protein; unassigned ortholog | duox1 |
| W4XKI6 | Phospholipid scramblase; unassigned ortholog | Sp-Plscr1 |
| W4YPA7 | Uncharacterized protein; unassigned ortholog | FLNA |
| Q05634 | Fascin; FSCN1; unassigned ortholog | FSCN1 |
| W4XHC0 | Tubulin beta chain; TUBB; unassigned ortholog | LOC586940 |
| W4ZFJ5 | ADP-ribosylation factor 1; ARF1; unassigned ortholog | LOC582325 |
| W4ZCC9 | Sodium/potassium-transporting ATPase subunit alpha; ATP1A4; unassigned ortholog | LOC404637 |
| W4Y5R7 | Integrin beta; ITGB; unassigned ortholog | LOC753206 |
| W4ZFV6 | Uncharacterized protein; unassigned ortholog | Sp-Rab5 |
| W4Y969 | actin CyI, cytoplasmic; ACTB; unassigned ortholog | LOC592912 |
| W4Z8A7 | 6-phosphogluconate dehydrogenase, decarboxylating; unassigned ortholog | Sp-Pgd |
| W4XAW2 | Guanine nucleotide-binding protein subunit beta; GNB; unassigned ortholog | LOC590203 |
| W4YP78 | ATP synthase subunit alpha; unassigned ortholog | LOC373382 |
| W4ZDE4 | Sodium/calcium exchanger 3; unassigned ortholog | LOC590169 |
| W4YY90 | Tubulin alpha chain; unassigned ortholog | Sp-Tuba1c |
| W4Y0E3 | Uncharacterized protein; unassigned ortholog | HSPA8 |
| W4YTE5 | Uncharacterized protein; unassigned ortholog | rab7 |
| A0A0B4J2V0 | Ras homolog gene family, member C-like; unassigned ortholog | LOC585398 |
| W4YCD5 | Uncharacterized protein; unassigned ortholog | Sp-Gi |
| A0A0B4J2W0 | Cell division cycle 42, GTP binding protein, 25kDa-like; CDC 42; unassigned ortholog | LOC584477 |
| W4ZBK1 | Histone H4; unassigned ortholog | Sp-EhH4_35 |
| W4XC29 | similar to Rab11 protein; unassigned ortholog | LOC763540 |
| H3IYA6 | Histone H3; unassigned ortholog | Sp-HH3.3 |
| W4Y4X0 | Ras-related protein; RAB1; unassigned ortholog | RAB1 |
| P16890 | Late histone H2B.L4; Fragment; unassigned ortholog | LOC767529 |
| W4XYP1 | Uncharacterized protein; unassigned ortholog | Sp-Hsp702A |
| W4YXN9 | Gelsolin-like protein 1; GSNL1; unassigned ortholog | LOC579255 |
| W4YM26 | Sorting nexin; SNX1; unassigned ortholog | LOC575614 |
| W4YUG2 | Ras-related protein; Rab-2A; unassigned ortholog | LOC577200 |
| W4XK99 | 14-3-3 epsilon; YWHAE; unassigned ortholog | LOC581376 |
| W4Z340 | 14-3-3-like protein; unassigned ortholog | LOC583263 |
| W4XN05 | Rab GDP dissociation inhibitor; GDI1; unassigned ortholog | LOC591552 |
| W4XQT8 | Ras-related protein; RAP1A; unassigned ortholog | LOC580151 |
| W4ZH65 | Uncharacterized protein; unassigned ortholog | Gp96 |
| Q5EAJ7 | Major vault protein; MVP ortholog | mvp |
| W4YKU8 | Similar to H(+-transporting ATPase; unassigned ortholog | LOC586747 |
| W4X9T4 | Tropomyosin; TPM; unassigned ortholog | LOC576168 |
| W4YFX5 | ADP/ATP translocase 3; unassigned ortholog | LOC575225 |
| W4YPT6 | Uncharacterized protein; unassigned ortholog | SPU_017814tr |
| W4YS38 | Malate dehydrogenase; MDH; unassigned ortholog | LOC587163 |
| W4YAH5 | Arylsulfatase; ARS; unassigned ortholog | LOC575079 |
| W4Z4Y0 | Uncharacterized protein; unassigned ortholog | Sp-Echn11 |
| H3IH34 | probable pterin-4-alpha-carbinolamine dehydratase; PCBD; unassigned ortholog | LOC594614 |
| W4XEK3 | Pyruvate kinase; PKM; unassigned ortholog | LOC763735 |
| W4XNS9 | Flotillin; FLOT; unassigned ortholog | LOC586881 |
| W4Y5V8 | Annexin; ANX; unassigned ortholog | LOC593594 |
| W4Y5X1 | Glucose-6-phosphate isomerase; GPI; unassigned ortholog | LOC762939 |
| W4YB86 | Glutamate dehydrogenase; GLUD; unassigned ortholog | LOC754749 |
| W4YDE0 | Adenosylhomocysteinase; AHCY; unassigned ortholog | LOC574714 |
| W4YDI9 | Uncharacterized protein; unassigned ortholog | Sp-Eno1 |
| W4YFN2 | similar to Epb7.2-prov protein; unassigned ortholog | LOC590338 |
| W4YM59 | similar to V-ATPase subunit A; unassigned ortholog | LOC589143 |
| W4YU75 | Scavenger receptor cysteine-rich protein variant 2; unassigned ortholog | LOC373213 |
| W4YV42 | Flotillin-1; FLOT1; unassigned ortholog | LOC586889 |
| W4YYF1 | Uncharacterized protein; unassigned ortholog | Sp-Dysf_1 |
| W4YYT1 | F-actin-capping protein subunit beta; CAPZB; unassigned ortholog | LOC579500 |
| W4Z7R9 | Annexin; unassigned ortholog | LOC590651 |
| W4ZCZ4 | Thymosin beta; THYB; unassigned ortholog | LOC373484 |
| W4ZD41 | Actin-5C; ACT5C; unassigned ortholog | LOC581650 |
| W4ZGC9 | Voltage-dependent anion-selective channel protein 2; VDAC2; unassigned ortholog | LOC576005 |
